# Scenario-based analysis of the impacts of lake drying on food production in the Lake Urmia Basin of Northern Iran

**DOI:** 10.1038/s41598-022-10159-2

**Published:** 2022-04-14

**Authors:** Bakhtiar Feizizadeh, Tobia Lakes, Davoud Omarzadeh, Ayyoob Sharifi, Thomas Blaschke, Sadra Karimzadeh

**Affiliations:** 1 Applied GISciences Lab, Department of Geography, Humboldt-Universität zu Berlin, Berlin, Germany; 2 Department of Remote Sensing and GIS, University of Tabriz, Tabriz, Iran; 3 IRI THESys, Humboldt-Universität zu Berlin, Berlin, Germany; 4 Graduate School of Humanities and Social Sciences, Hiroshima University, Hiroshima, Japan; 5 Department of Geoinformatics, University of Salzburg, Salzburg, Austria; 6 Remote Sensing Laboratory, University of Tabriz, 5166616471 Tabriz, Iran

**Keywords:** Environmental chemistry, Environmental impact, Climate sciences, Environmental sciences, Endocrinology

## Abstract

In many parts of the world, lake drying is caused by water management failures, while the phenomenon is exacerbated by climate change. Lake Urmia in Northern Iran is drying up at such an alarming rate that it is considered to be a dying lake, which has dire consequences for the whole region. While salinization caused by a dying lake is well understood and known to influence the local and regional food production, other potential impacts by dying lakes are as yet unknown. The food production in the Urmia region is predominantly regional and relies on local water sources. To explore the current and projected impacts of the dying lake on food production, we investigated changes in the climatic conditions, land use, and land degradation for the period 1990–2020. We examined the environmental impacts of lake drought on food production using an integrated scenario-based geoinformation framework. The results show that the lake drought has significantly affected and reduced food production over the past three decades. Based on a combination of cellular automaton and Markov modeling, we project the food production for the next 30 years and predict it will reduce further. The results of this study emphasize the critical environmental impacts of the Urmia Lake drought on food production in the region. We hope that the results will encourage authorities and environmental planners to counteract these issues and take steps to support food production. As our proposed integrated geoinformation approach considers both the extensive impacts of global climate change and the factors associated with dying lakes, we consider it to be suitable to investigate the relationships between environmental degradation and scenario-based food production in other regions with dying lakes around the world.

## Introduction

The importance of large lakes as significant freshwater resources is widely acknowledged^1^. Large lakes provide recreational, economic, and ecosystem services to millions of inhabitants worldwide. Global climate change and associated environmental changes have significantly impacted many lakes in recent decades. Many well-known lakes are shrinking or drying up, and some lakes are even dying. Some noteworthy examples include Lake Chad, the Sea of Galilee, and Lake Urmia^2^. Lake drying can lead to major environmental and socioeconomic problems such as water scarcity, land degradation, and food shortages. Lakes around the world have experienced at least four periods of significant shrinkage due to climatic changes in the last 5 million years. Between 7.65 million and 7.9 million years ago, lake water levels dropped by up to 250 m^3^.

Among other effects, the drying up of lakes can significantly impact Food Production (FP). Given the potential impacts of the rapid rates in global population growth and the projected effects of environmental changes on FP, the United Nations (UN) and local governments increasingly acknowledge the need to address food security as one of the major challenges to be faced in the coming decades^4^. According to the UN^5^, the world population will reach about 9.7 billion by 2050. While this will increase the global demand for food resources, global environmental changes are expected to negatively impact the food production capacity^6^. Addressing FP in the context of lake drying is particularly important as the land degradation of the lake ecosystems could have ramifications for peace, quality of life, and food security^7^ (see Table 1 for a full list of acronyms). When dying lakes lead to and/or intensify salinization (e.g., Lake Urmia, Iran), the environmental and social impacts on FP are more significant and obvious.

**Table 1:** Acronyms.

FP	Food production
UN	United Nations
LUB	Lake Urmia Basin
LUCC	Land use/cover changes
AAT	Average annual temperature
AAP	Average annual precipitation
CSRI	Combined Spectral Response Index
SAR	Synthetic aperture radar
PCA	Principal component analysis
WQI	Water Quality Index
MCDA	Multi criteria decision analysis
FANP	Fuzzy analytical network analysis
DST	Dempster Shafer theory
FOBIA	Fuzzy object based image analysis
FSE	Fuzzy synthetic evaluation

Lake drying could pose significant challenges for the provision of reliable, affordable, and nutritious food sources required for maintaining the health and wellbeing of the global population. Accordingly, the main goal of this research is to apply an integrated geoinformation approach to examine the FP in environments affected by drying lakes. We focus on Lake Urmia as a case study. The proposed integrated geoinformation approach could support international and local decision-makers and authorities in developing efficient policies for mitigating the impacts of climatic changes on the fragile ecosystems of drying lakes. The results of this study and application of the proposed approach to other major dying lakes could inform the development and implementation of action plans that can, ultimately, contribute to preventing large-scale food crises and human suffering^8^.

### The case of Lake Urmia

Lake Urmia is one of the most hyper-saline and well-known drying (and even dying) lakes. It has been drying up since 2000. The lake, at an elevation of 1286 m above mean sea level and with an area of 5000 km^2^, is located in north-western Iran (Supplementary Fig. 1). Lake Urmia and its watershed are critical in the daily life of millions of people as well as 226 species of birds and other animals. The Lake Urmia Basin (LUB), with an area of 51,876 km^2^, hosts agricultural areas for FP and industrial activities. Extensive plains and fertile agricultural farmlands surrounding the lake have made LUB one of the most critical areas for food production and animal husbandry in Iran. According to the Iranian Ministry of Agriculture^9^, with about 500,000 hectares** of farmlands (360,000 croplands and 140,000 orchards), the LUB accounts for 8.4% of the total area of farmland in Iran. There are 42 cities and 520 villages in the LUB, according to the National Statistics Center of Iran^10^. Based on the latest Iranian census (2016), 7.3 million people live in the LUB, which is about 9.2% of Iran’s total population. Due to climate change and intensive land use/cover changes (LUCC), the lake has lost about 65–85 percent of its surface area since 2000, increasing the expanse of flat salty areas^2^. Aside from changing climate conditions, inappropriate irrigation practices and extensive anthropogenic pressure are further factors contributing to the drying up process, leading to drastic changes to the ecosystem of the LUB. The gradual drying up of the lake and associated environmental changes are also anticipated to lead to salt and dust storms, and extensive soil and water salinization. Such major environmental issues will threaten public health and the FP in the coming decades. From the environmental perspective, it is believed that a reduction in the lake’s water level will lead to the formation of salt domes and intensify desertification, thereby threatening the productivity of the nearby farmlands through soil and water salinization. As with other drying lakes, there is also great concern that the lake will completely dry up in the future (see Supplementary Fig. 2 as an example).

### Climate change impacts

**Figure 1:**
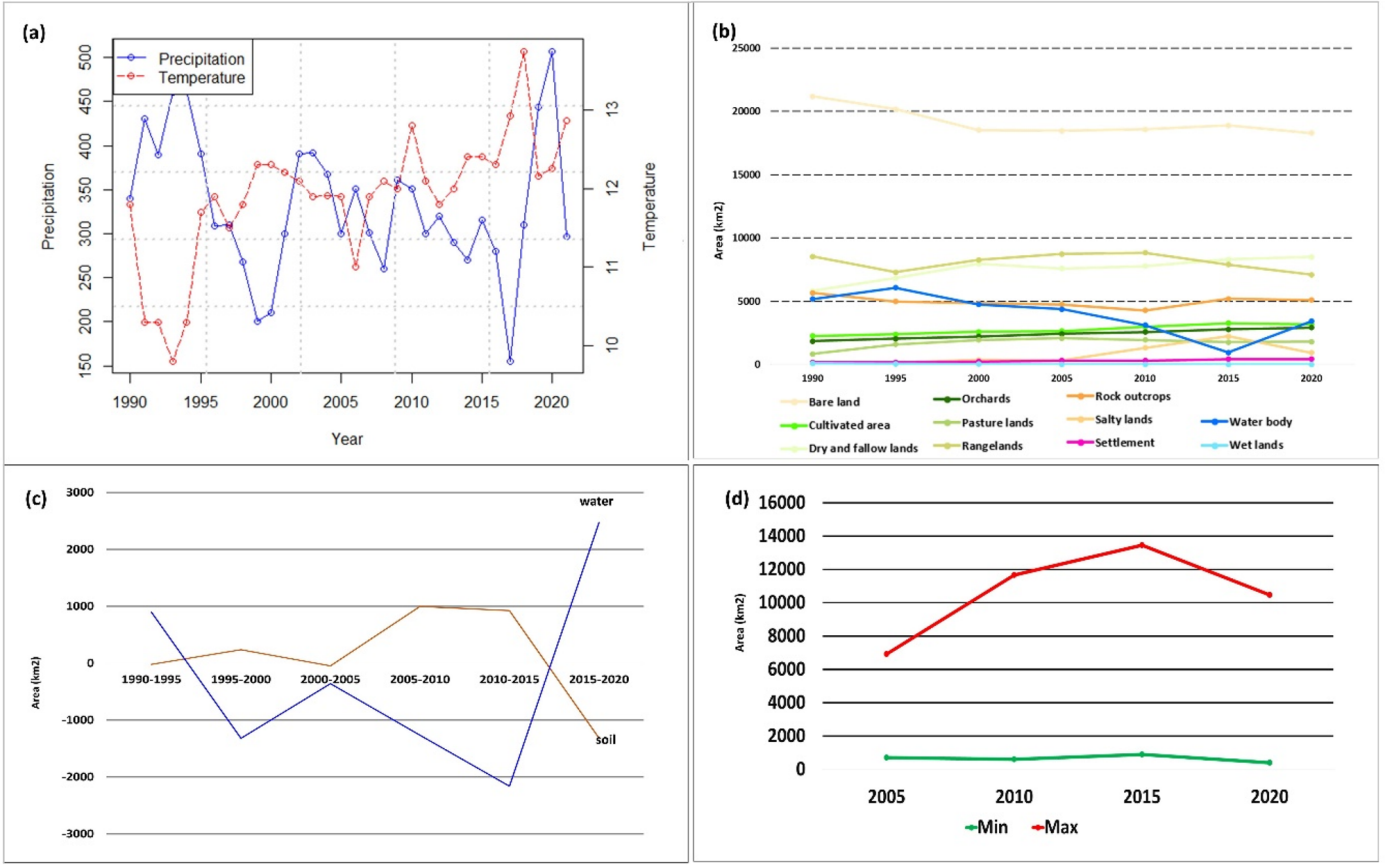
(**a**) Average annual precipitation and an average annual temperature time series s, indicating the climate change impacts on the lake drought, (**b**) land use/cover change in the LUB derived from time series image analysis from 1990–2020, (**c**) time series analysis of changes in the soil salinity and water body of the lake and the extension of soil salinity in the past three decades, and (**d**) time series assessment of groundwater salinization from 2000 to 2020.

**Figure 2:**
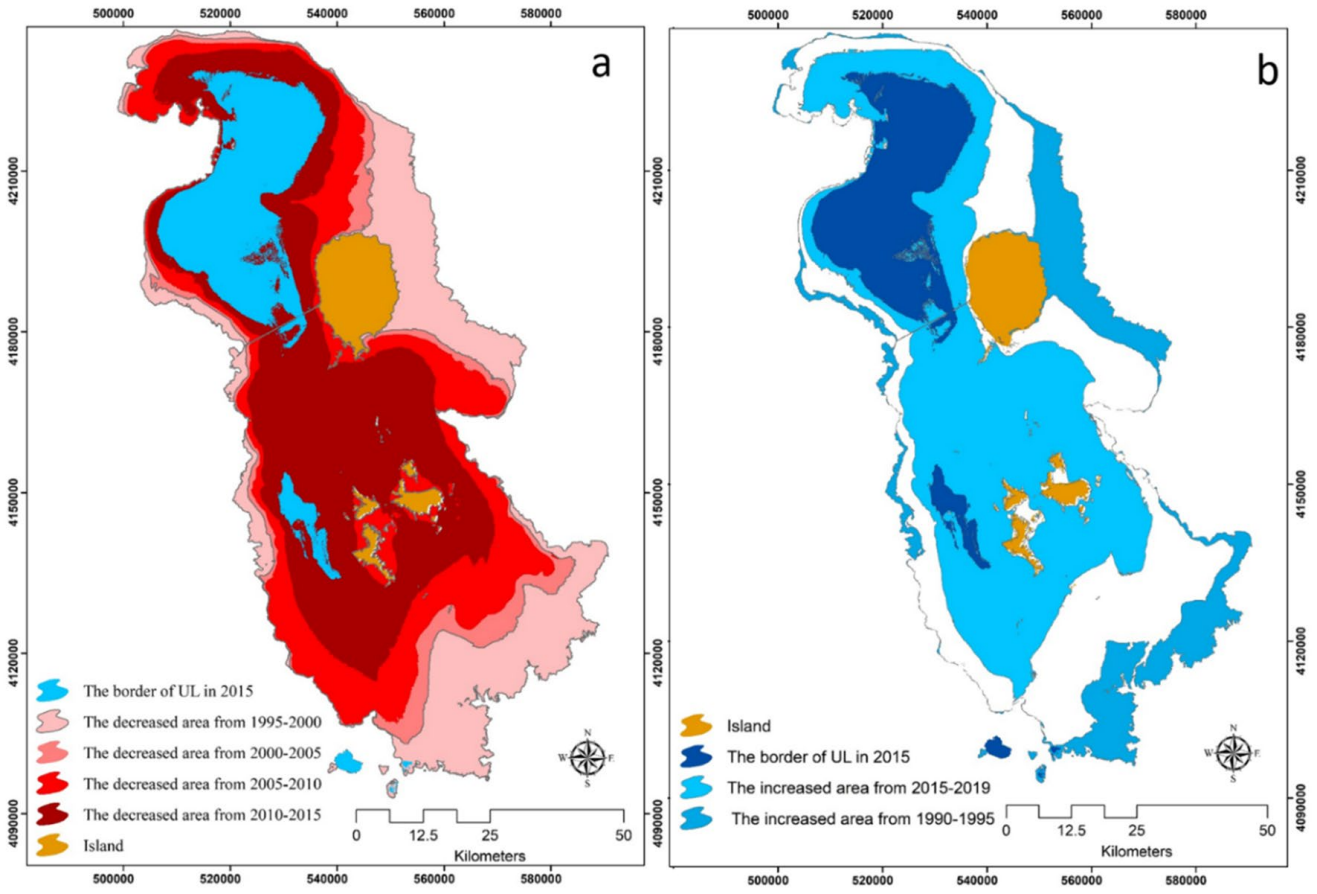
Observed changes of the Urmia Lake area from 1990 to 2020 based on the satellite images: (**a**) shows how the area of the lake has decreased over time, and (**b**) shows the periods of increased water body from 1990–1995 and 2015–2019 based on the increase the annual precipitation. Figure created in Arc GIS 10.7 ESRI, https://www.esri.com/.

The impact of climate change on FP is one of the most threatening environmental challenges for today's societies. The LUB, with a semi-arid climate, an average annual precipitation (AAP) of 350 mm (mm) and an average annual temperature (AAT) of 12.5 °C, has been experiencing an intense drought over the past decades^11–16^. Figure 1a represents the AAP and AAT trends for the past 30 years in the LUB. As this figure shows, there were several stages of drought, especially from 1995 (465 mm) to 1998 (268 mm). There was a slight increase in the AAP between 2000 (210 mm) and 2002 (390 mm). While the general drought continued until 2017 when the LUB received less than 155.47 mm AAP, the AAP increased between 2018 (309.75 mm) and 2020 (507.1 mm), which led to an increase in the lake’s surface area for two consecutive years. The figures also show a considerable AAP decrease in 2021. The AAT trends show an increase of about 2 °C between 1995 (11.7 °C) and 2018 (13.7 °C). The spatial distribution of the temperature at the basin level shows that the drought of the lake has a direct spatial correlation with the temperature distribution, whereby the temperature around the lake increases as the lake continues to shrink. Figures 2a,b show the impacts of climate change on the water body of Lake Urmia between 1990 and 2020. As this figure shows, the lake's size reduced significantly between 2005 and 2015, when it lost about 80% of its area (Fig. 2a). As Fig. 2b shows, there was a slight increase in surface area due to the increasing AAP from 2018 to 2020. Other factors, such as increased awareness about the critical condition of the lake and the implementation of several policies to limit the expansion of farmlands and prohibit the cultivation of high-water demand crops in the LUB may have also contributed to this increase in the water body of the lake. However, despite this slight increase of the lake’s water body, our investigation indicated that, overall, the lake has still lost 1766 km^2^ (34.5%) of its surface area between 1990 and 2020, resulting in intensive environmental issues in LUB.

### Anthropogenic pressure

**Figure 3:**
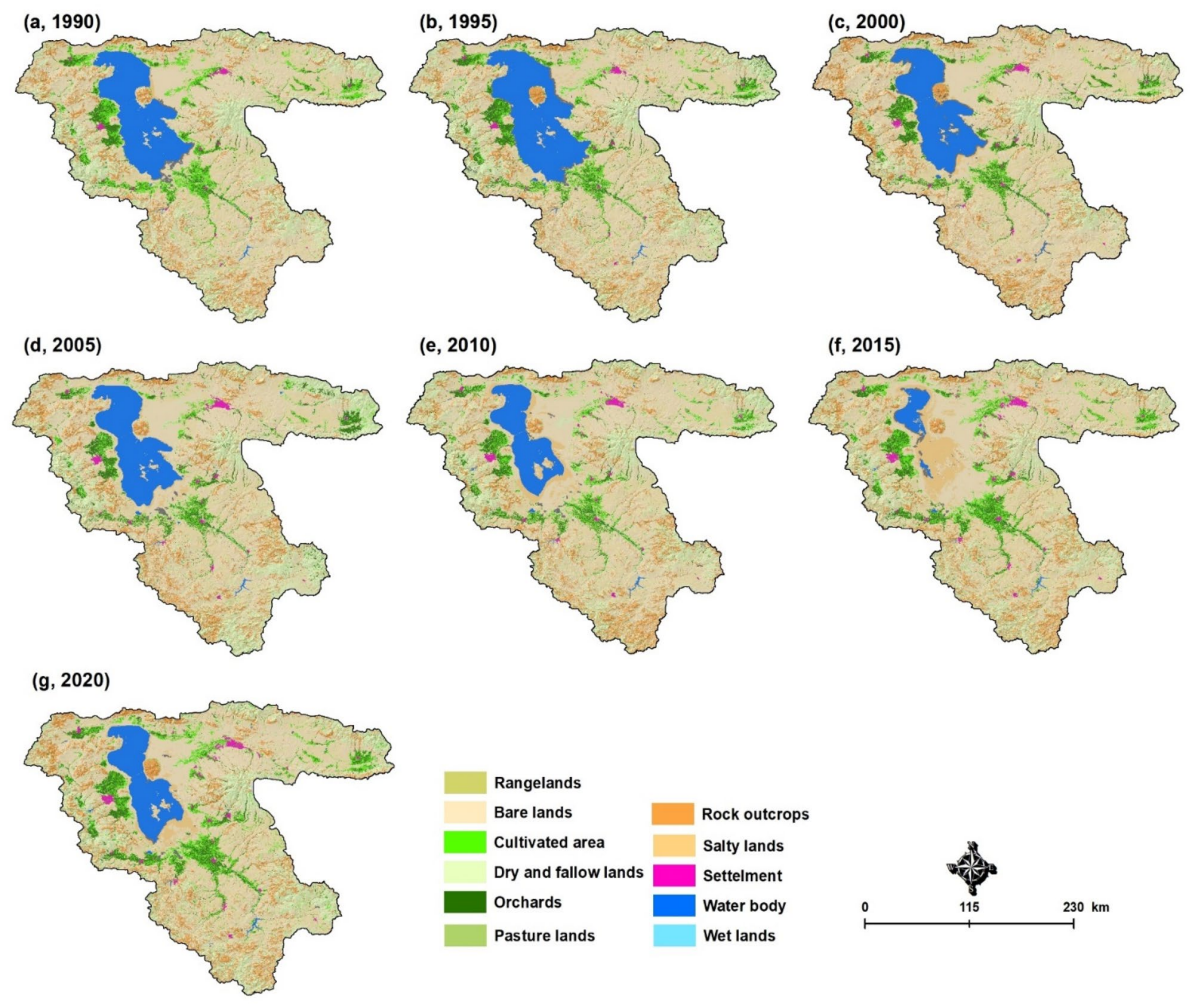
Results of the land use/cover changes monitoring and time series assessment in the Lake Urmia basin from 1990–2020, which show the increase in the area of farmlands, and the reduction of the rangelands over the past three decades. Figure created in Arc GIS 10.7 ESRI, https://www.esri.com/.

In the past two centuries, rapid industrial development and increasing resource demands have led to major land use/cover changes (LUCC), land degradation, and deforestation in many parts of the world. Understanding the spatiotemporal pattern of LUCC provides critical information for more efficient management practices towards sustainable development and FP^17^. In addition, intensive LUCC can negatively impact the environment and socioeconomic conditions^18^. In most drying lake ecosystems, the insufficient and irrational LUCC cause a variety of environmental pressures and issues that further complicate global crises such as food insecurity^19^. In the context of LUB, the results of this study indicate that extensive LUCC have occurred in the past three decades. Figure 3 represents the LUCC maps for LUB derived from satellite images using image processing methods. According to our results, the area of croplands and cultivated areas significantly increased from 2005 to 2015, which contributed to the Lake Urmia drought by increasing the water demand and extraction from the nearby aquifers for farmland irrigation. It is worth mentioning that the agriculture system in Iran is essentially based on the traditional irrigation systems of flood/surface irrigation, which require large amounts of water. In addition, farmers around the lake mainly produce high-water demand plants and crops such as onions, tomatoes, potatoes, sugar beets, grapes, apples, peaches, and nectarines, which has also contributed to the lake drought. The changes between 1990 and 2020 indicate an intensive growth of the settlement areas, dry and fallow lands, and orchards. At the same time, the rangeland areas and rocky outcrops decreased. Based on these maps, the LUCC were computed to affect about 2,000,000 hectares, which is equivalent to 47.1% of the LUB. The main change was observed to be in the rangelands (− 70.1%). In the second and third rank, the orchards with + 41.2% and the croplands with + 36.6% have also experienced considerable changes in area (Fig. 1b).

### Land degradation

**Figure 4:**
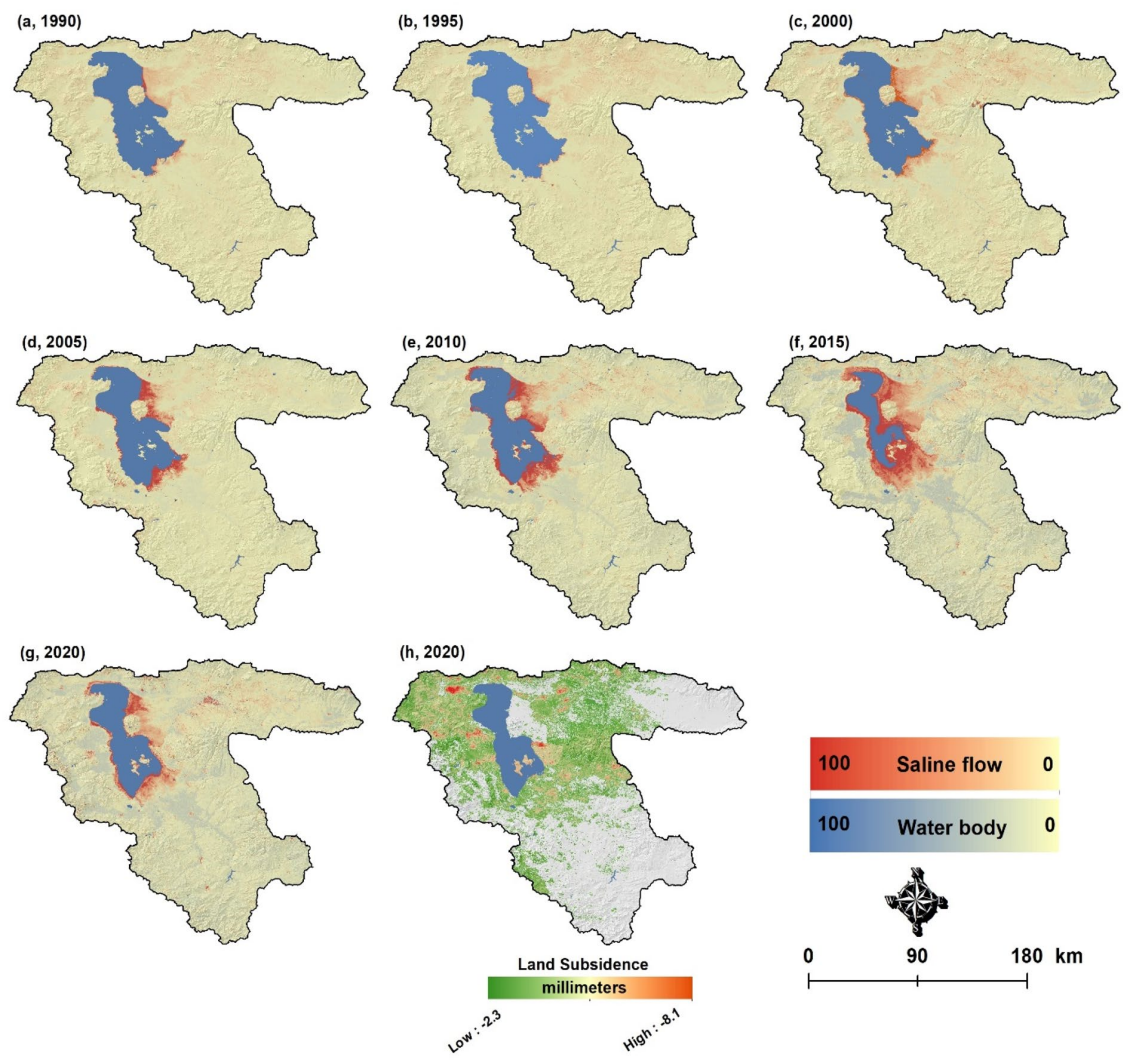
Results of the soil salinization from 1990–2020 (**a**–**g**) and land subsidence from 2915–2020 (**h**). Red indicates the propagation of saline flows that have significantly impacted the productivity of farmlands. Figure created in Arc GIS 10.7 ESRI, https://www.esri.com/.

Land degradation is one of the unavoidable consequences of lake drying, especially in the LUB^2^. In this study, the soil salinization and land subsidence were monitored to detect the impacts of the Lake Urmia drought on the land degradation of the surrounding areas. Therefore, the soil salinization from 1990 to 2020 was evaluated using earth observation satellite images. The resulting soil salinity maps based on the Combined Spectral Response Index are presented in Fig. 4. As this figure shows, the salty lands/deserts and soil salinity in the LUB have significantly increased in the eastern area surrounding the lake. The area of the salty lands and salinization continuously increased from 1995 to 2015, with the most significant increase in 2015 when the lake reached its worst critical condition. Figure 1c also shows the trend of the water body and soil salinity for the study period and the spatial correlation between the lake drought and the extension of soil salinity. From the hydrodynamic perspective, it must be indicated that the elevation of the lake bed increases from west to east, which means that the water depth in the eastern area of the lake is much less than in the western areas. Thus, the lake generally shrinks from east to west. In addition to climate change and intensive LUCC, the causeway that divides the lake into north and south has changed the balance of water around the lake. The causeway was constructed to connect Tabriz and Urmia, two major cities that are located on the western and eastern sides of the lake, respectively. The causeway has reduced the normal water circulation by about 48–50%. As a result, the salt density in the northern part and the concentration difference between the two parts have increased by about 49%, which is another environmental change that has exacerbated the lake drought and intensified the associated environmental impacts^20^.

Land subsidence is another aspect of land degradation and a critical issue in LUB. Land subsidence occurs when large amounts of groundwater are withdrawn from certain types of rocks, such as fine-grained sediments. The ground compacts after the water is removed, which can lead to collapse^21^. We evaluated the land subsidence in LUB for the 2015 to 2020 timeframe based on the available Synthetic Aperture Radar (SAR) dataset. Figure 4h compares the 2015 and 2020 SAR images used to compute the land subsidence ratio in the LUB. Based on the results, the maximum and minimum land subsidence velocity in the study area were − 2.26 mm/year and − 8.13 mm/year, respectively. The number of legal and illegal wells in the LUB was estimated to be 87,242. The spatial distribution of subsidence areas shows a meaningful correlation with the density of the excavated wells in the western and eastern plains of Lake Urmia^22^. This implies that water extraction for drinking and agricultural- and industrial demands play an essential role in the progressive land subsidence in the study area. Patterns of intensive land subsidence have also been reported by other studies in different parts of the LUB^23–26^ (see Supplementary Fig. 3 as an example).

### Groundwater salinization

**Figure 5:**
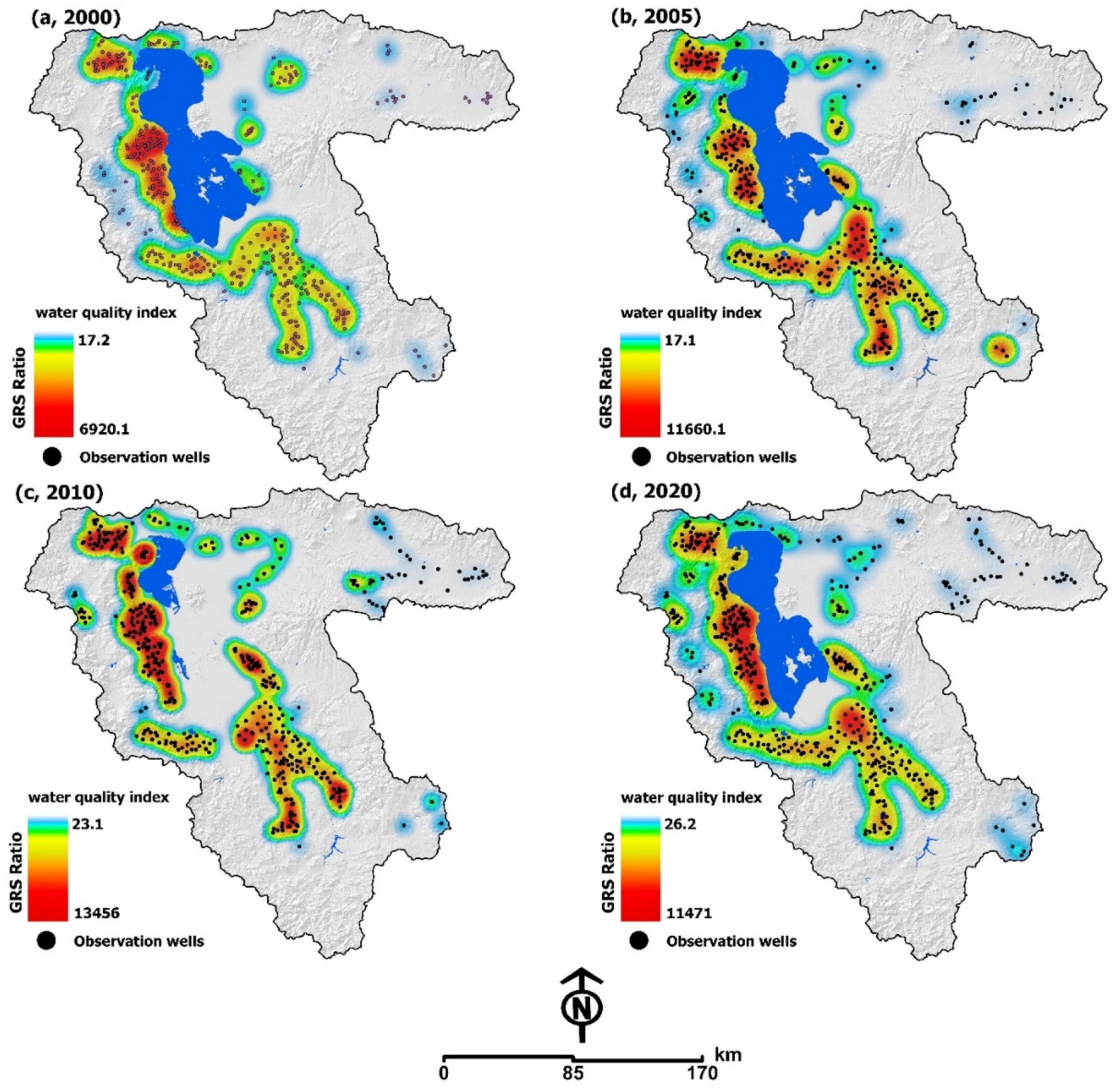
Results of monitoring the trend of groundwater resources salinization (GRS) and the way it has spread to the farmlands (red colors) due to the over discharge of aquifers. Figure created in Arc GIS 10.7, ESRI, https://www.esri.com/.

Earlier research indicated that the intensive groundwater extraction resulted in a negative balance in the aquifer. Nowadays, the tangible results of this can be observed as groundwater salinization, and land subsidence take hold in the LUB^21–27^. Based on the groundwater level simulation, the intensive groundwater extraction for agricultural and industrial activities has resulted in at least 5–15 m of groundwater drawdown, which is a decline of about 50–60% over the past three decades^28–30^. Figure 5 shows the results of spatiotemporal groundwater quality modeling for 2000–2020 based on available monitoring data, including samples and chemical analysis, from 856 wells in the LUB. Results show that the extraction from adjacent aquifers has changed the groundwater level balance and increased the interaction of saltwater and fresh groundwater. The groundwater quality in the surrounding area, especially in the southern and western areas, has been impacted by the hyper-salinity of the lake water and the processes of saltwater encroachment and intrusion (Fig. 1d). The excessive discharge of aquifers and the disruption of the groundwater resources can supplant the interphase threshold and lead to saltwater intrusion into adjacent aquifers, which is also acknowledged by previous studies^30^ (see Supplementary Figs. 4 and 5 for spatial distribution of the wells and trend of groundwater discharge).

The anthropogenic sources of contaminants, such as chemical fertilizers, industrial waste, and untreated sewage water, might also be significant factors contributing to the excessive degradation of the groundwater quality. The principal component analysis (PCA) identified two main factors in almost all the aquifers and three main factors in the shore of the lake that explained more than 80% of the total variance. From a hydrodynamic perspective, it can be assumed that, based on the hyper-saline nature of Lake Urmia, the total hardness and salinity of the groundwater is related to the interaction of the salt water and freshwater aquifers and the dissolution of bedrock material, which are the dominant processes affecting the groundwater quality in the surrounding areas. The degradation can result from a combination of natural and anthropogenic processes, but these can be closely related. Based on the water quality index (WQI) values, computed to assess the groundwater quality of the LUB for drinking water purposes, approximately 48% of the groundwater samples were identified to have poor quality and be unsuitable for drinking and agricultural purposes, according to the World Health Organization standards^31^. Based on this, we conclude that the combined approach of a multivariate statistical technique and spatial analysis is effective in helping us understand the factors determining the groundwater quality. According to the results, the WQI has essentially increased from 2000 to 2015 (6920.06, 11,660.09 and 13,456.02), indicating a reduction in the quality of water used for drinking and agricultural activities. However, from 2015–2020 there is a slight reduction (11,470.8), which may be attributed to the increase in the AAP and the policies for controlling the groundwater extraction as part of Lake Urmia Restoration Program. The WQI analysis results illustrate the impacts of the hyper-saline water and the distribution of the salt from the lake bed to the farmlands, which makes them unsuitable for FP.

### Prediction of the environmental impacts of lake drought on FP

**Figure 6:**
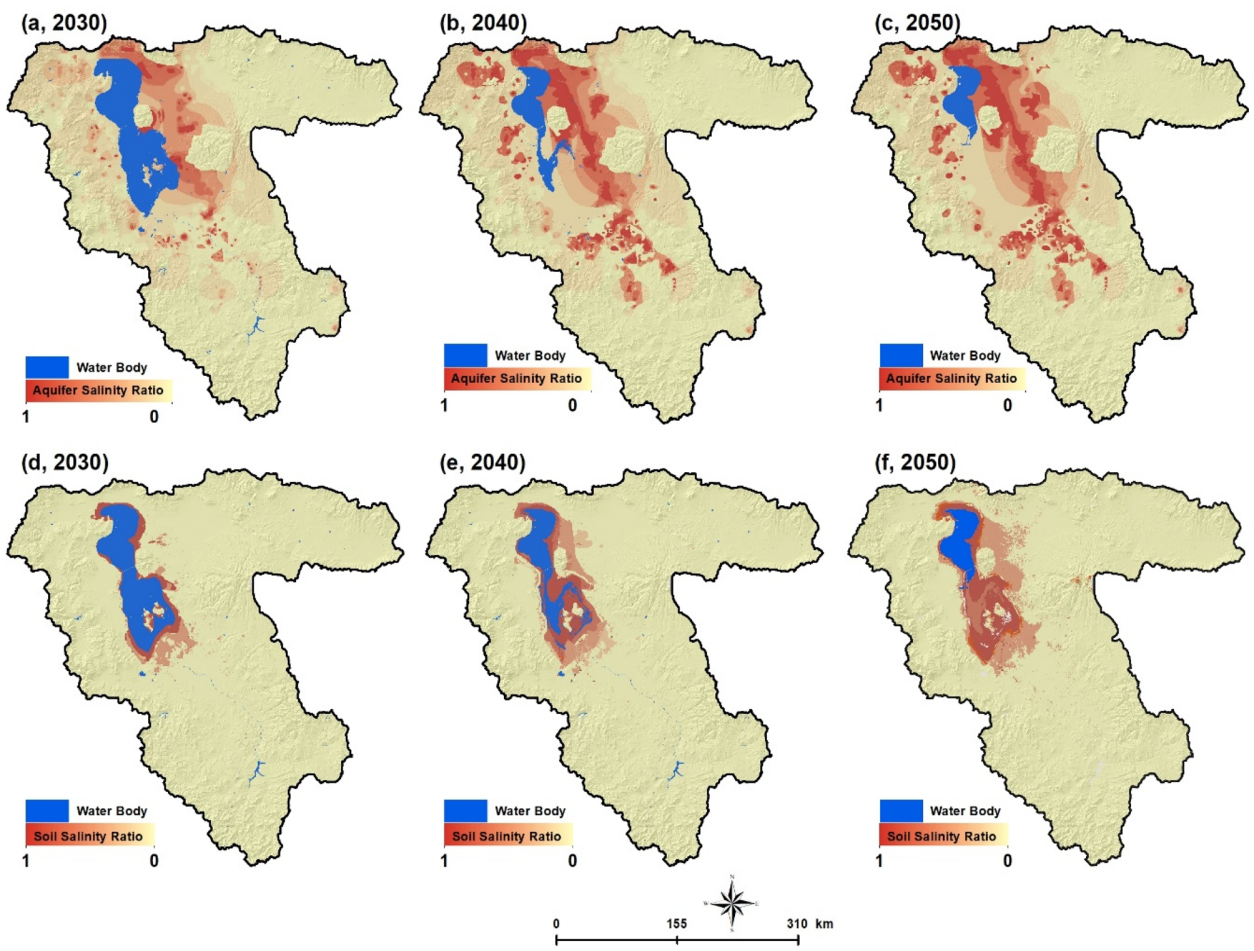
Results of simulation using a CA-Markov: aquifer salinization for 2030 (**a**), 2040 (**b**), 2050 (**c**) and soil salinization for 2030 (**d**), 2040 (**e**) and 2050 (**f**). Red color indicates the extension of soil and aquifer salinization and significant limitation for food production. Figure created in Arc GIS 10.7, ESRI, https://www.esri.com/.

After identifying the trend of environmental changes from 1990 to 2020, we identified the current limitations and opportunities for FP in the LUB. Soil and aquifer salinization forecasting maps can be used to optimize management practices and prevent FP from being compromised in the future. We used the CA-Markov method (see “Methods” section) to predict the future development for the years 2030, 2040 and 2050 (Fig. 6). As Fig. 6 shows, the Lake Urmia drought will continue in the coming decades, and by 2050 only the northern part of the lake will remain while all other parts will be covered by salt and dust. As shown in Fig. 6a–c, the groundwater quality will be fundamentally impacted by the lake saltwater intrusion due to the extensive water extraction from the aquifers. The groundwater salinization will impact almost all aquifers around the lake, which will, in turn, intensify the water scarcity and affect the water supply for drinking as well as for industrial- and agricultural activities. Figure 6d–f also show the predicted soil salinization resulting from the lake drought. Based on these maps, the soil salinization will extend to the eastern and southern parts of LUB where the most productive farmlands are located and produce several million tons of food annually (see Supplementary Table 1).

**Figure 7:**
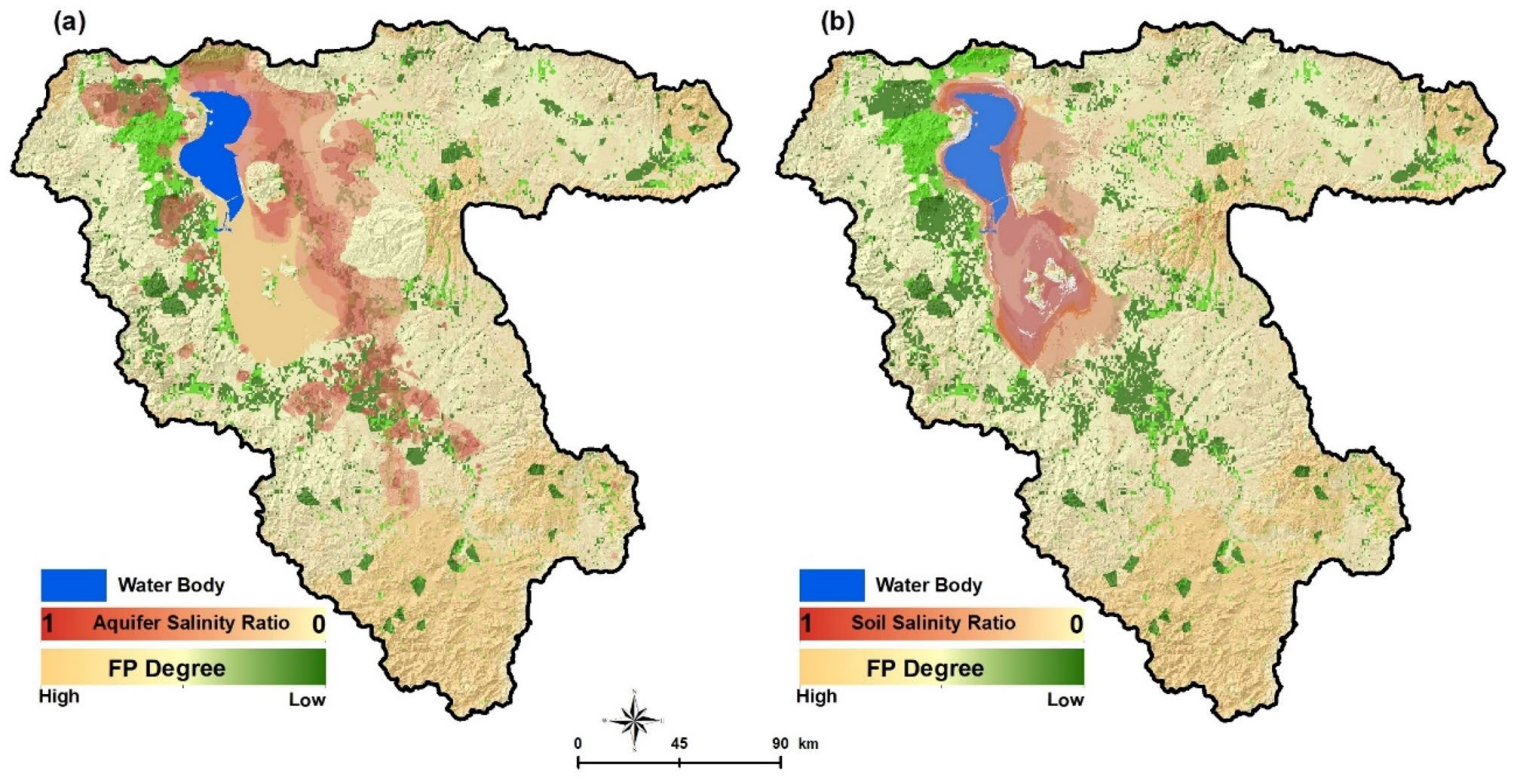
Results of the scenario-based food production for 2050 and its spatial correlation with aquifer salinization (**a**) and soil salinity (**b**). The FP degree was computed based on the CA-Markov method. The green color shows the farmlands and red color indicates how the expansion of the soil and aquifer salinization has impacted the productivity of the farmlands. Figure created in Arc GIS 10.7 ESRI, https://www.esri.com/.

**Figure 8:**
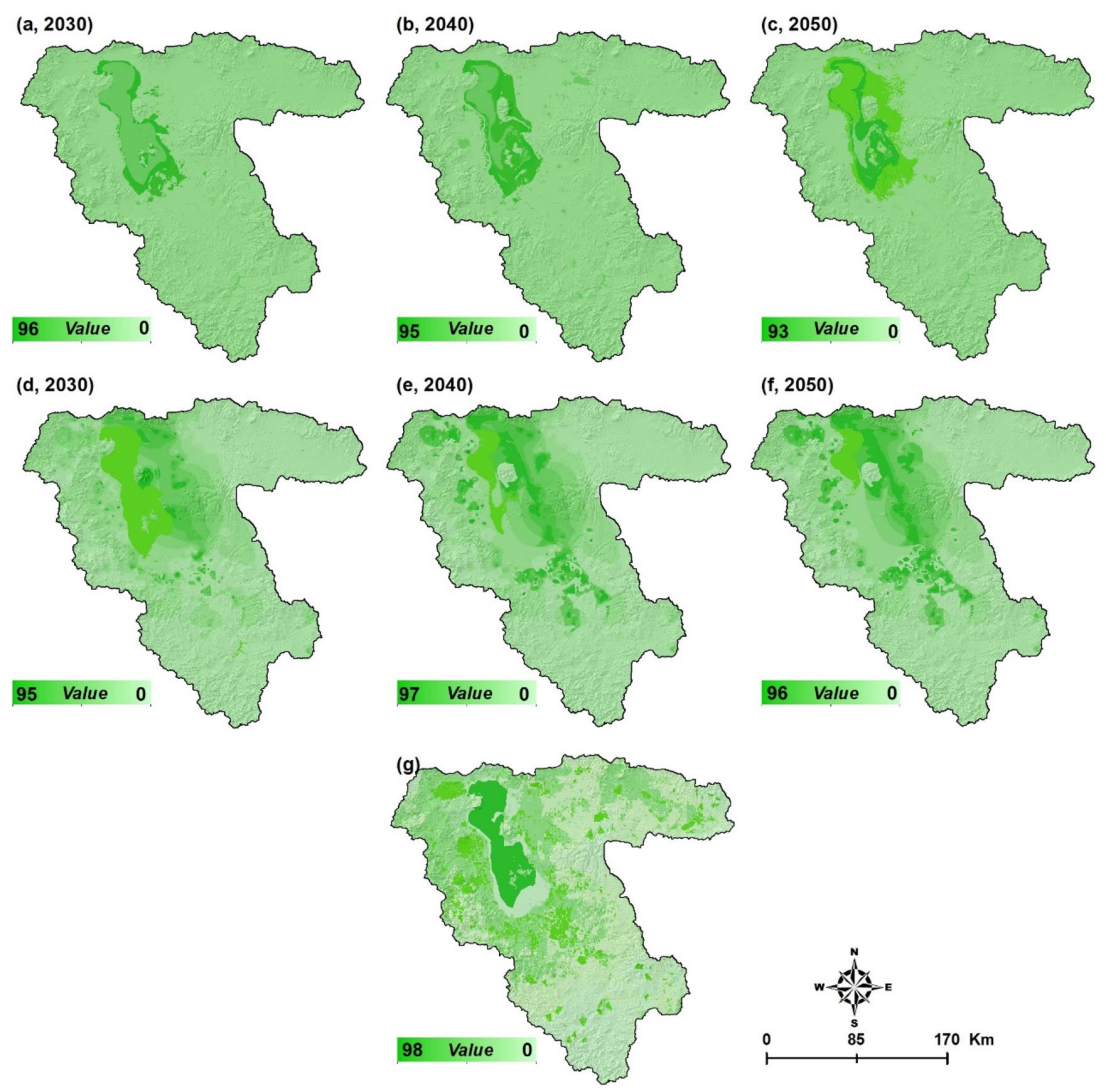
Results of the spatial uncertainty analysis based on the DST for the predicted soil salinization (**a**–**c**) and groundwater salinization (**d**–**f**), and the food production degree map that was generated based on the CA-Markov method (**g**). The values from 0 to 100 show the confidence level and the reliability of results based on the DST technique. Figure created in Arc GIS 10.7 ESRI, https://www.esri.com/.

Based on the climate change trend, land degradation, water salinity, LUCC, and environmental issues, we developed FP scenarios for the LUB using a GIS-based multiple spatial analysis to prioritize and compute the degree of sustainability of agricultural farmlands under the impacts of the lake drought. Figure 7 shows the computed spatial distribution of the results of the scenario-based FP analysis. The productivity of the agricultural lands is expected to be reduced substantially due to the impact of soil and aquifer salinization. Results of this simulation indicated that the farmland areas for FP are likely to be reduced significantly by both soil and aquifer salinization. Table 2 represents the computed farmland areas expected to be impacted by both aquifer and soil salinization. The farmlands of LUB cover an area of about 500,000 hectares, of which about 375,000 hectares currently contribute to FP according to the environmental and land suitability assessment (Fig. 7). Based on the simulation, about 29,100 hectares and 20,600 hectares will be impacted by aquifer and soil salinization, respectively. These numbers are anticipated to increase to 248,000 and 52,000 hectares by 2040. The simulation shows even worse environmental conditions for 2050 when about 260,200 hectares (69.4%) of highly productive farmland will be affected by aquifer salinization. It is also anticipated that by 2050 132,420 hectares (35.4%) of the highly productive farmlands will be directly impacted by soil salinization and lose their fertility and, thus, suitability for crop development. In addition, land subsidence will also increase significantly and affect the infrastructure and farmlands. Scenario-based results indicate that the LUB is going to face critical environmental conditions. While the LUB area currently produces 8.4% of the total food produced in Iran and feeds 7.3 million people, it is now clear that the region will face severe challenges in the coming decades. Figure 8 shows the results of the spatial uncertainty analysis for the predicated soil salinity and groundwater salinization for 2030, 2040 and 2050, as well as the FP degree computed based on the CA-Markov method. The computed maps represent high levels of confidence, which confirm the validity of the results.

**Table 2:**
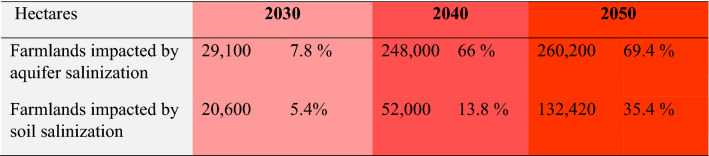
Results of scenario-based Lake Urmia drought impacts on the productivity of farmlands in LUB. Numbers and percentages indicate aquifer and soil salinization impacts on the current farmlands, with an area of about 375,000 hectares.

## Discussion

The results of this research indicate that degrading environmental conditions in the LUB will significantly impact the FP. Our investigations show that mismanagement of water resources, the extension of the agricultural farmlands, and the excessive water extraction from aquifers have contributed to the lake drought and have led to problems, such as extensive land degradation and soil- and water salinity, which threaten the FP. The lack of sustainable development strategies and the mismanagement of the fragile ecosystem has contributed to these problems over the past three decades. Thus, plans and programs based on a sustainable development agenda are urgently needed to mitigate the anticipated food security crisis and associated socioeconomic and environmental problems. Such plans and programs should be designed and implemented according to the scientific evidence of environmental and socioeconomic factors and under consideration of the local characteristics of the LUB. They should be based on specific, realistic, measurable, practical, and time-bound actions to bring about actual changes. It is also critical to develop decision-making approaches that can be optimized and improved by establishing feedback mechanisms to determine the valuable insights from unfulfilled outcomes^31^.

According to the results of our land use/cover mapping and field work, the agriculture system in the LUB is characterized by family farming (in small land lots) and is sustained based on the traditional irrigation system. A wide variety of high-water demand crops (e.g., onions, tomatoes, potatoes, watermelons, grapes, etc., see Table 1 in the Supplementary Appendix for a complete list of the crops) have been produced on this basis over the past decades based on farmers' choices and market demand. Promoting precision agriculture with low water demand crops could be a great opportunity to counteract the lake drying and, at the same time, maintain agricultural productivity. The modification of cropping patterns towards low water demand crops could be a cheap and efficient solution for reducing the overall agricultural water consumption, but this requires verification through future studies in this area. The main objective of this study was to analyze the environmental impacts of the Urmia Lake drought on food production. The obtained results are intended to be employed as input for future policy making and analysis programs. While crop modification to suit the environmental conditions and water availability may be an effective option for reducing the over-use of aquifers, this suggestion is based on the opinion of the authors and the feasibility of this idea must be verified by future studies that consider the specific aspects of ecology, land suitability, and socioeconomic implications to select the appropriate low water demand crops and promote their adoption in the area. In addition, potential land readjustment and aggregation of small farms, through participatory and democratic approaches, to develop a modern agriculture system would benefit farmers and reduce the irrigation water demand. Developing a land suitability analysis under specific policy making programs for low water demand and horticultural crops shall support decision-makers and authorities in the agriculture sector in deciding on the presence or absence of a specific plant in the optimal cropping pattern^32^. It should, however, be mentioned that a transition to horticulture requires dedicated upgrades to the existing infrastructure (including roads and cold chains) to ensure efficient delivery of products to consumers. The ULB is one of the major agro-industrial areas of Iran and has an efficient transport network that facilitates the development of a dedicated program for the mitigation of the lake drought impacts on FP.

Sustainability is one of the most critical global initiatives of the human lifetime^33^. Thus, considering the political circumstances (i.e., international sanctions), the rate of population growth, as well as the importance of the LUB for the national food production (8.4%) and the progressive land degradation and water shortage, plans and programs for sustainable development in the LUB are urgently needed. In accordance with other studies, we consider changes to the FP to be one of the most effective measures to mitigate the environmental impacts of lake drought. Sustainable agriculture development also requires suitable strategies for balancing the environmental, economic, and social dimensions of food and agriculture governance^31^. In this context, international policy guidelines such as ‘agenda 2030’, with its guidelines for ensuring FP in light of the increasing global environmental challenges, shall support the decision-makers in improving the environmental and socioeconomic status of the LUB^32^.

## Methods

In this research, we applied an integrated geoinformation approach comprising a fuzzy-object-based image analysis (FOBIA) and spectral analysis to obtain a LUCC and soil salinization time series. We used the SAR satellite images to detect the land subsidence from 2015 to 2020. We applied an integrated GIScience spatiotemporal analysis to analyze the trends of climate indicators, soil characteristics, and water resources to develop the food production map. The central aspect of the research methodology is explained in the following:

### Land use/cover mapping and trend assessment

We processed Landsat time series satellite images for LUCC and soil salinization monitoring. To detect changes, we obtained the satellite images for July of 1990, 1995, 2000, 2005, 2010, 2015 and 2020. To obtain the most accurate results, we applied the integrated FOBIA approach to LUCC monitoring and mapping. Therefore, we applied a multi-resolution segmentation to obtain image objects. For a FOBIA classification, segmentation parameters are critical since they directly impact the size of image objects and the final classification results. We estimated the scale parameter^34^ and performed the segmentation with a scale of 20, a shape index of 0.6, and a compactness value of 0.4. We identified the primary land use/cover pattern of the LUB based on field work and a comprehensive discussion with the local authorities and decision-makers. We employed a FOBIA classification to derive the characteristics of the LUCC subclasses. The training data were collected in field operations and from existing and historical LUCC and cadaster maps and aerial photography, which were provided by the Agricultural Resource Organization. We used a total of 21,000 points (3000 points for each study year) to identify the characteristics of each LUCC subclass and as training data for the FOBIA classification. We were able to identify the relevant object features based on the spectral and spatial characteristics of each LUCC subclass and evaluated their effectiveness for deriving each LUCC subclass by comparing the training data with the fuzzy membership value of each object feature based on the following equations:

- Spectral attributes/brightness
  1 $${\varvec{B}} = \frac{1}{{{\text{n}}_{{{\text{vis}}}} }}{ }\mathop \sum \limits_{{{\text{i}} = 1}}^{{{\text{n}}_{{{\text{vis}}}} }} {\overline{\text{c}}}_{{{\text{i}}\left( {{\text{vis}}} \right)}}$$
  where *B* is the mean brightness of an object and ${{ \overline{\text{C}}}}_{{{\text{i}}\left( {{\text{vis}}} \right)}}$ is the sum of all the mean brightnesses in the visible bands divided by the corresponding number of bands n_vis_.
- Normalized Difference Vegetation Index (NDVI)
  Tv = mean NDVI
  2 $${\text{f }}\left( {{\text{Object}}} \right) = \left\{ {\begin{array}{*{20}l} { \text{LC}\quad \text{ if f}\left( {{\text{Object}}} \right) \le {\text{T}}_{{\text{v}}} )} \\ {\text{VA}\quad \text{if f}\left( {Object} \right) > {\text{T}}_{{\text{v}}} )} \\ \end{array} } \right.\quad {\text{T}}_{{\text{v}}}^{{\prime}} = \frac{{{\text{meanNDVI}}_{{\text{LC }}} + {\text{meanNDVI}}_{{{\text{VA}}}} }}{2}$$
  $T_{v}^{{\prime}}$ is an average of the mean NDVI values for the candidate object of LUCC class and vegetated areas (VA). The NDVI, which has a value between − 1.0 and + 1.0
  Green Normalized Difference Vegetation Index (GNDVI)
  3 $${\text{GNDVI}} = 100*\left( {{1} + \left( {\left( {\left[ {\text{Mean band 4}} \right] - \left[ {\text{Mean band 2}} \right]} \right)/\left( {\left[ {\text{Mean band 4}} \right] + \left[ {\text{Mean Layer 2}} \right]} \right)} \right)} \right)$$
- Modified Normalized Differenced Water Index (MNDWI)
  4 $${\text{MNDWI}} = 100*\left( {{1} + \left( {\left( {\left[ {\text{Mean Band4}} \right] - \left[ {\text{Mean Band5}} \right]} \right)/\left( {\left[ {\text{Mean Band4}} \right] \, + \left[ {\text{Mean Band5}} \right]} \right)} \right)} \right)$$
- Soil Water Content Index (InfraRed Index)/ Soil Color Index
  5 $${\text{SWCI }}\left( {{\text{IR}}} \right) = \, \left( {{\text{NIR}} - {\text{ ETM7}}} \right)/\left( {{\text{NIR}} + {\text{ETM7}}} \right)$$
  6 $${\text{SCI }} = {\text{ R }} - {\text{ G }}/{\text{ R}} + {\text{G}}$$
- Normalized Built-up Index (NDBI)
  7 $${\text{NDBI}} = \, \left( {\left[ {\text{Mean band 5}} \right] - \left[ {\text{Mean band 4}} \right]} \right)/\left( {\left[ {\text{Mean band 5}} \right] + \left[ {\text{Mean band 4}} \right]} \right)$$

Salinity Index (SI)

- Normalized Difference Salinity Index
  8 $$\text{Salinity Index (SI)} = ([ \text{Mean Layer }1]*[ \text{Mean Layer }3])^{0.5}$$
  9 $${\text{NDSI}} = \left( {\left[ {\text{Mean band 3}} \right] - \left[ {\text{Mean band 4}} \right]} \right)/\left( {\left[ {\text{Mean band 3}} \right] + \left[ {\text{Mean band 4}} \right]} \right)$$
- Shape Rectangular
  10 $$1 - \sqrt {\frac{{{\uplambda }_{{{\text{min}}}} }}{{{\uplambda }_{{{\text{max}}}} }}}$$
  ${\uplambda }_{{{\text{min}}}}$ is the minimal eigenvalue. ${\uplambda }_{{{\text{max}}}}$ is the maximal eigenvalue
  11 $$\frac{{\left\{ {\# \left( {x,y} \right)\varepsilon P_{v} :\rho v\left( {x,y\rho } \right) \le 1} \right\}}}{{\# P_{v} }}P_{v} = \left( {{\text{x}},{\text{y}}} \right){\text{ is the elliptic distance at a pixel }}\left( {{\text{x}},{\text{y}}} \right)$$
  [0,1]; where 1 is a perfect rectangle.
- Shape Index
  12 $$\frac{{{\text{B}}_{{\text{v}}} }}{{4\sqrt {\#_{{\text{V}}} }_{{\text{v}}} }}$$
  ${\text{B}}_{{\text{v}}}$ is the image object border length
  13 $$4\sqrt {\#_{{\text{p}}} }_{{\text{v}}} {\text{is the border of square with area of}}\, \# P_{v}$$

After identifying the relevant object-based features for LUCC classification, we exported the computed membership values for all objects in LUB to Python for the deep learning classification and to produce the final LUCC map. To validate the results, we applied an accuracy assessment based on the fuzzy synthetic evaluation (FSE), which was proposed by Feizizadeh^35^ and acknowledged for its effectiveness in FOBIA classifications by several studies^33–35^. Technically, the FSE makes use of two sets of data, namely reference data (ground truth data) and the results of the OBIA-based classification map. The FSE is used to compute the confidence ratings, which give the classification confidence and observed level of error for each class^36^ (see Supplementary Table 2). 30% of all ground control points (6300 out of 21,000) were used as references data, and the overall accuracy for the study period of 1990–2020 with 5-year intervals was computed to be 0.94, 0.93, 0.98, 0.95, 0.96, 0.93 and 0.97, respectively (see Supplementary Table 3 for results of accuracy assessment based on the FSE).

### Land degradation monitoring (soil salinity and land subsidence)

We used the obtained Landsat images to carry out a LUCC classification to monitor and map soil salinization. According to earlier studies, the spectral properties of Landsat allow us to compute the soil salinization rate based on the soil salinity indices^37,38^. The soil characteristics are impacted by various factors (e.g., vegetation cover, moisture, texture, parent material, etc.), which must be considered in soil salinity mapping^39,40^. Therefore, we applied the Combined Spectral Response Index (CSRI) technique to compute the soil salinity ratio for each study year. The CSRI method was developed by Fernandez-Buces et al.^41^ and is an efficient method for determining soil salinity^41–43^. The CSRI is based on the spectral information of RGB and the near-infrared bands, and it is computed as follows:

14 $${\text{CSRI}} = \frac{{{\text{B}} + {\text{G}}}}{{{\text{R}} + {\text{NIR}}}} \times {\text{NDVI}} {\text{ and }}\left( {{\text{B}} + {\text{G}}} \right)/\left( {{\text{R}} + {\text{NIR}}} \right) \times {\text{NDVI}}$$

NIR: Near-infrared and NDVI: Normalized Differences Vegetation Index.

We used the field observation data together with the soil electrical conductivity (SEC) data to validate the results. Time series SEC data were obtained from the Agricultural Resource Organization. In order to validate the results, a linear regression analysis was applied to compute the spatial correlation between the reference data and the results of the CSRI method for each study year. The result of the linear regression analysis from 1990–2020 in 5-year intervals was computed to be 0.93, 0.92, 0.90, 0.92, 0.96, 0.97 and 0.95, respectively, which represents a very high spatial correlation between the reference data and the obtained soil salinization maps.

We also monitored the land subsidence in the LUB. Therefore, we applied differential interferometric synthetic aperture radar analysis (DInSAR) for the 2015–2020 period to create a land subsidence map. The DInSAR technique is a reliable and fast approach to derive long- and short-term deformations. The technique mainly relies on the ‘master’ and ‘slave’ SAR image processing of the same part of the earth from the same satellite orbit. In the repeated pass, the difference of phase value correlation of two SAR images can be used to estimate the ground subsidence. Generally, the phase correlation or coherence ($\gamma$) of two acquisitions in DInSAR analysis contains various sources. After reducing non-deformation phases, such as the atmospheric phase, topographic phase, and noises, the multi-temporal DInSAR can be implemented. In the multi-temporal analysis, images are taken at different times (T_1_, T_2_, …, T^n^), and the phase value of the image is a simple way to measure the changes in satellite-to-target direction (R_1_, R_2_, …,R_n_) across the same study area^44–46^. For the DInSAR analysis, we used 6 single look complex (SLC) images of descending orbits (T 79) of Sentinel-1 to cover the study area. The images were taken on the 2015.11.11, 2017.11.12, and 2020.11.02, and cover the upper and lower parts of the Lake Urmia Basin (6 images in total) in interferometric wide (IW) mode. The images are in VV (vertical–vertical) and VH (vertical–horizontal) polarizations, and the incidence angle of the images is 39.08°. The incidence angle differences of the first pair (2015.11.11 and 2017.11.12) and the second pair (2017.11.12 and 2020.11.02) are 0.009° and 0.01°, respectively. Due to the semi-arid to arid climate in the study area, the large temporal baseline (~ 2 years) is not a serious problem for the interferometric analysis, as confirmed by similar studies^23–26^.

### Water salinization spatiotemporal analysis

The significance of freshwater for agricultural-, industrial-, and residential purposes is beyond debate. Due to the semi-arid climate in the LUB, agricultural activities were traditionally based on surface run-off water and groundwater. The LUCC results reveal a substantial increase in croplands and farmland over the past 30 years, which depend heavily on groundwater. The regional water organization has monitored the LUB water quality parameters annually since 2000. We made use of observations from deep- and semi-deep wells, springs, and Qanats during the rainy season. Since the number of observation wells has increased every year since the Lake Urmia drought, the groundwater observation data used in the spatiotemporal modeling of the groundwater quality assessment was limited to 630 in 2005, 684 in 2010, 751 in 2015, and 859 in 2020. We obtained and analyzed the following water quality parameters for the studied time period (2000–2020): electrical conductivity, power of hydrogen (pH), potassium (K^+^), total hardness, sulfate (SO_4_^2−^), magnesium (Mg^2+^), chloride (Cl^−^), sodium (Na^+^), total dissolved solids, calcium (Ca^2+^), carbonate (CO_3_^2−^), and bicarbonate (HCO_3_^−^). We obtained groundwater physicochemical data and evaluated the water status using the water quality index (WQI), Spearman correlation, principal component analysis (PCA), and agglomerative hierarchical clustering based on the GIS spatial analysis for determining the hydro-geochemical attributes. The statistical evaluation of the physicochemical groundwater parameters is essential to understand the main factors controlling water quality variations over time^47^.

Multivariate statistical techniques such as the PCA are effective data visualization and mining approaches. We applied a PCA technique to identify correlations between physical and chemical characteristics in the aquifer and to elucidate complicated patterns in data matrices^47,48^. Squared cosines with absolute values of more than or equal to 0.4 were used for the exploration of the observed ions^49,50^. We used the Spearman correlation coefficient to identify the sources of different elements in the groundwater samples. High coefficients reveal the significance of the relationship between two parameters. A positive coefficient indicates that the associated parameters are similar and harmonious, while a negative coefficient indicates that the variables are moving in opposite directions. We carried out an agglomerative hierarchical cluster analysis of the groundwater data to identify a specific pattern of similar observations within the studied variables^48^. The hierarchical clustering allowed us to identify suitable structures in chaotic and complex data and thus simplify the explanation of observations^51^. We obtained the distribution characteristics and created the groundwater quality maps using a GIS spatial analysis. While many interpolation methods are available, we choose to use the inverse distance weighted interpolation technique, which is the most frequently used deterministic modeling tool^52^. The model is based on the hypothesis that a node shares more similarities with nearby points and that it is influenced more strongly by the surrounding data values. We used 856 points and derived the node values by averaging their combined weighted total.

### Environmental food production mapping

We applied a GIS-based multi criteria decision analysis (GIS-MCDA) to multiple factors of FP in the LUB to examine food production under the impact of the Lake Urmia drought. Food production in a fragile ecosystem such as the LUB must consider the interaction of several causal spatial indicators. The GIS-MCDA is an effective approach for dealing with the spatial decision problems and patterns based on the concept of ‘spatial thinking’^53,54^. The GIS-MCDA allows us to consider the relevant spatial indicators and their characteristics as attributes, under consideration of the decision maker’s preferences, to analyze the spatial problems^54^. We analyzed food production in the LUB by considering relevant climate indicators affecting agricultural production, namely annual average precipitation, temperature, sunshine hours, and humidity. In the context of soil characteristics, the soil degradation, fertility, texture, and depth were also considered as indicators. From the land characteristics perspective, the salt scattering spots, and land use/cover were also included as relevant indicators for FP (see Supplementary Fig. 6). The selection of these indicators is based on expert opinions, data availability, and relevant research literature^31,54,55^.

After finalizing the environmental indicators, the initial data were collected from the relevant governmental departments, satellite images, and the Spatial Data Infrastructure (SDI) of LUB. All data were gathered, relevant geometric edits were applied, and the indicators were developed into a spatial GIS dataset. Since the spatial GIS data were collected from heterogeneous resources with different scales, we used the fuzzy standardization process to standardize the indicators in the same scale for criterion weighting. This approach considers the degree of fuzzy membership values on a scale of 0–1 based on the benefit/ cost context of the indicators^52^. In the GIS-MCDA analysis, the significance of each criterion is computed as part of the decision analysis. We employed the Fuzzy Analytical Network Analysis (FANP) to determine the significance of each indicator. The FANP is an efficient GIS-MCDA weighting tool^53^. We included the knowledge of 35 experts from different departments of the University of Tabriz and the University of Urmia. We asked these experts from agricultural-, natural resource-, and food security departments to initially rank the provided indicators. Then, based on the FANP method, we yielded the following criteria weights: precipitation (0.098), temperature (0.096), sunshine hours (0.075), humidity (0.058), groundwater depth (0.071), water quality (0.095), soil degradation (0.069), soil fertility (0.098), soil texture (0.099), soil depth (0.098), salt scattering spots (0.086), and land use/cover (0.098). The FANP compression matrix is provided in Supplementary Table 4. For the criteria weighting, it is necessary to compute the consistency ratio (CR) to validate the obtained weights. According to Saaty^56^, a CR < 0.1 indicates an acceptable level of consistency among the experts and that the results can be employed for a spatial aggregation. Our study yielded an acceptable CR value of 0.065.

Criteria weighting in GIS-MCDA significantly influences the uncertainty and reliability of the results^57^. Therefore, we applied a global sensitivity analysis (GSA) to determine the validity of the computed weights using the FANP method. The GSA approach is used to compute the two critical indexes of *S* (first order) and *St* (total effect). The letters *S* and *St* denote that the FANP's weights were assigned in a semantic manner. Any discrepancy in the value and order of the S and St indices, as well as the reference weights (e.g., FANP's weights), can be regarded as uncertainty associated with the criteria weights^58^. Accordingly, we used a ‘weighted overlay’ spatial aggregation function to produce the food production capability map.

### Environmental prediction based on the CA-Markov

We used a combination of a Markov model and a Cellular Automaton model (CA-Markov) to predict the land degradation and water salinity based on the trend observed in the past 3 decades (1990–2020). Both techniques are considered effective for GIScience prediction problems^59^. A typical CA–Markov model separates the discrete cellular, finite state, neighbor, and rule features into four categories while analyzing the trend as follows:

15 $$Z_{{\left( {I + 1} \right)}} = Z_{\left( I \right)} \times Q$$

Z(I) = the first map in year *A l, Z*(*l* + *1*) = the second map year *B, l* + *1, Q* = state transition matrix, and Z can be described as the following matrix:

16 $${\text{Z}} = \left[ {\text{Z1 Z2 Z3}} \right]$$

*Zi *(*i* = 1, 2, 3) represents the changes between the classes of map *A* and *B, Q* can be described as an [*n, n*] matrix in the following, where *n* = total number of changes between map *A* and *B, Qij* = transition probability for any changes between map *A* and *B* from the timeline of *i* to *j*, and the sum of each row of the matrix should be equal to 1.

17 $$Q = \left[ {\begin{array}{*{20}l} {Q_{11} } &\quad {Q_{12} } &\quad \ldots &\quad {Q_{1n} } \\ {Q_{21} } &\quad {Q_{22} } &\quad \ldots &\quad {Q_{2n} } \\ \vdots &\quad \vdots &\quad \vdots &\quad \vdots \\ {Q_{n1} } &\quad {Q_{n2} } &\quad \ldots & \quad {Q_{nn} } \\ \end{array} } \right],\quad \mathop \sum \limits_{{\left( {j = 1} \right)}}^{n} Q_{ij} = 1,{ }1{ } \le i,{ }j{ } \le n,{ }n = 4$$

According to a transformation function, the next state cell is decided by the current state and its surroundings^59,60^. This method is based on the generation of a transition probability matrix between two maps in different timelines. The transition probability matrix enables an assessment that indicates the likelihood of each pixel in class A of the first map class converting to another class (e.g., B, C, D, …) or remaining in class A in the second map class^61^. The Markov chain, which is technically a separate random process, uses transition probability to forecast the next state and all future states based on the current state^62^. Except for some of the after-effect occurrences, it is a viable method for predicting regional characteristics. We used CA-Markov to predict the land use/cover, soil, and water salinization maps for 2030, 2040, and 2050 based on the trend obtained for each environmental indicator.

### Spatial uncertainty analysis

Understanding the spatial uncertainty is critical in geospatial analysis and modeling. Data quality, correctness, inaccuracy, vagueness, fuzziness, and imprecision are all referred to as spatial uncertainty in GIScience^35^. Uncertainty in GIS-based modeling is inevitable due to the variety caused by a heterogeneous dataset, expert opinions, and model error, which has forced the GIScience community to develop spatial and statistical approaches to understand and quantify uncertainty^62^. The Dempster Shafer Theory (DST) is an efficient technique for modeling the imprecision and computing the spatial uncertainty^62^. It is based on mathematical operations employing Bayesian probability theory^2^. The DST can be used to compute the epistemic uncertainty that affects expert knowledge of the probability P (M_) within the alternative model M_, = 1,…,n. This is also known as the ‘theory of evidence’, which aims to compute the BBA m (A) on sets A (the focal sets) of the power set P(Z) of the event space Z, i.e., on sets of outcomes rather than single elementary events^62^. The belief function is used to compute the lower limit value for a (known) probability to determine the spatial uncertainty. The plausibility function additionally predicts the upper bound value for an (unknown) probability. The geographic uncertainty can be calculated using the difference between the plausibility (*Pl*) and belief (*Bel*) functions. We employed the *Belief* function in Idrisi software to calculate DST and to define the spatial uncertainty of the predicted soil and water salinization maps for 2030, 2040, 2050, as well as the computed food production map (see Fig. 8).

18 $$m:{ }\;\;P(Z) \to \left[ {0,1} \right],\;\;m(0) = 0;\quad \mathop \sum \limits_{A \in (Z)}^{ } m(A) = 1$$

## Supplementary Information

Supplementary Information.

## Data Availability

The data that support the findings of this study are available on request from the corresponding author.
