# Supplementary material for: Scenario-based analysis of the impacts of lake drying on food production in the Lake Urmia Basin of Northern Iran
Source: Sci Rep. 2022 Apr 14;12:6237. doi: 10.1038/s41598-022-10159-2 (PMC9010442; doi:10.1038/s41598-022-10159-2)
Supplement: Supplementary file 1 — Supplementary Information. [file 41598_2022_10159_MOESM1_ESM.docx]

**Supplementary materials for:**

**Scenario-based analysis of the impacts of lake drying on food production in the Lake Urmia Basin of Northern Iran**

Bakhtiar Feizizadeh^[[1]](#footnote-1)ab^, Tobia Lakes^bc^, Davoud Omarzadeh^a^, Ayyoob Sharifi^d^, Thomas Blaschke^e^, Sadra Karimzadeh^a^

^a^ Department of Remote Sensing and GIS, University of Tabriz, Iran

^b^ Applied GISciences lab, Department Geography, Humboldt University of Berlin, Germany

^c^IRI THESys, Humboldt-Universität zu Berlin, Germany

^d^Graduate School of Humanities and Social Sciences, [Hiroshima University](https://www.google.com/url?q=https%3A%2F%2Fwww.hiroshima-u.ac.jp%2Fen%2Fgshs&sa=D&sntz=1&usg=AFQjCNGTgYrbj01uEsvRj_uUWMulbJK0uw), Japan

Department of ^e^Geoinformatics, University of Salzburg, Austria


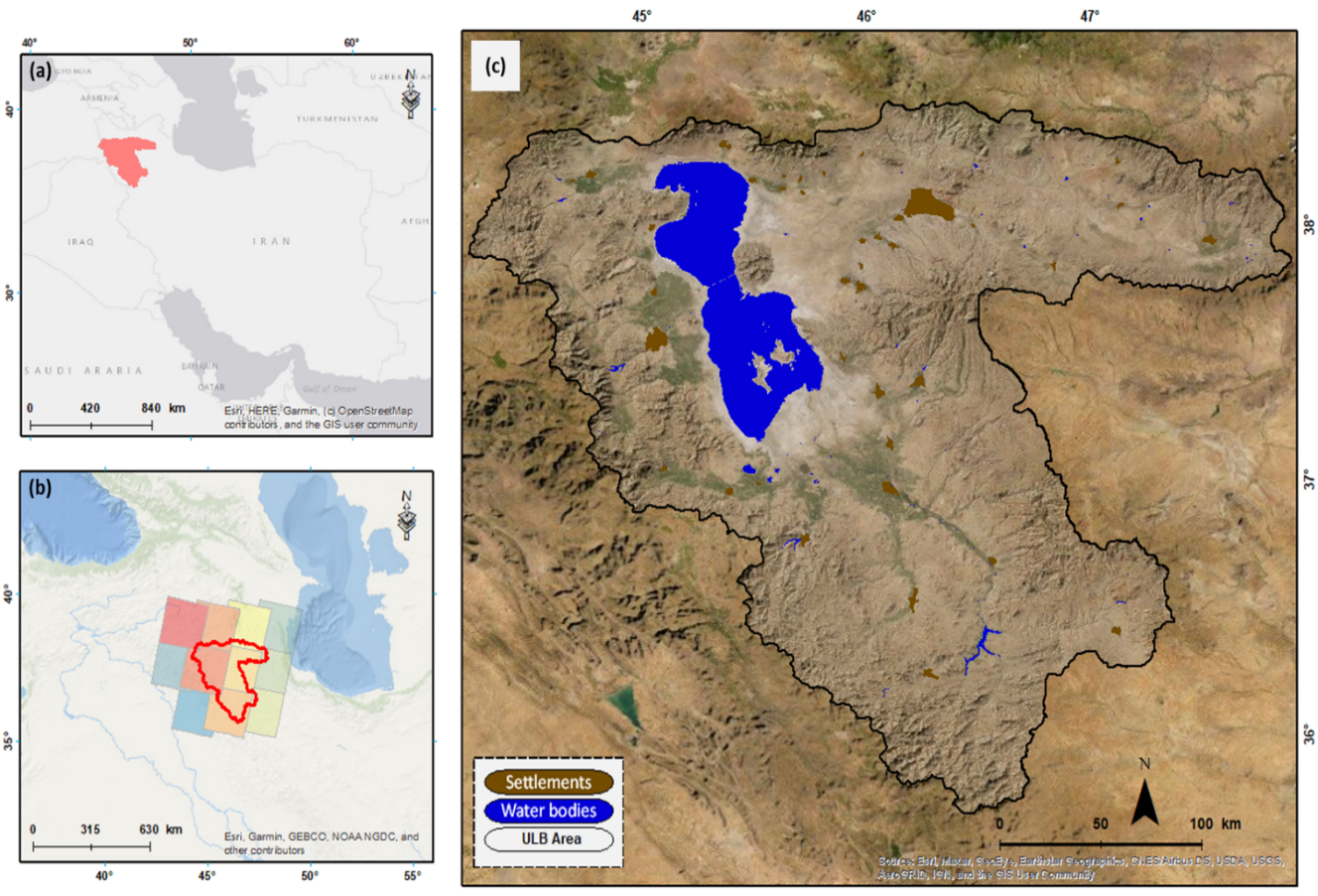


Supplementary **Fig.1**. Location of the Lake Urmia in Iran


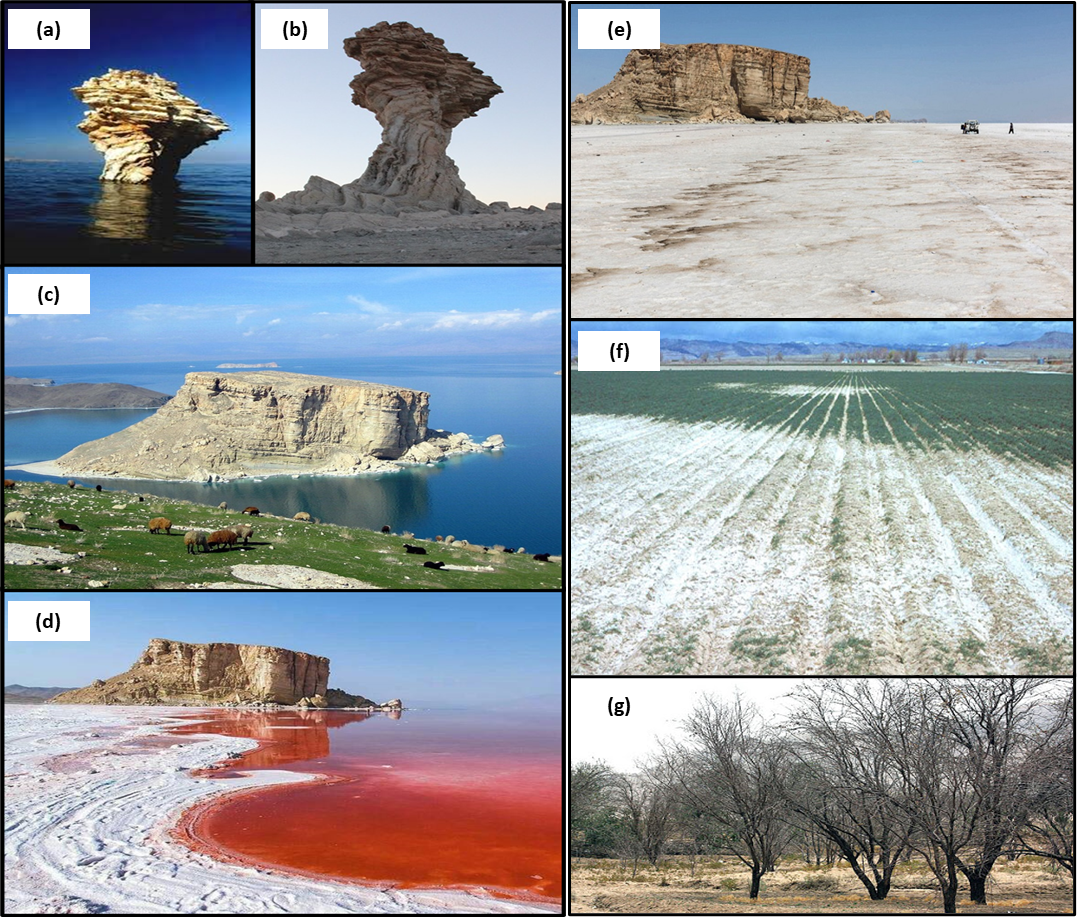


Supplementary **Fig.2**. Environmental changes associated with the Urmia lake drought in 1995 and 2011 before and after drought: (a, b), before and after condition of the famous “Osman” island; (c, d, and e), before and after condition of the famous “Kazimdashi” island; (f) an example of the effect of salt release on farmlands; and (g) an example of the (i) influence of the drought of Urmia Lake on orchards. As it can be observed from these figures, the lake drought has led to serious environmental issues such as soil salinization, thereby negatively affecting agricultural activities.


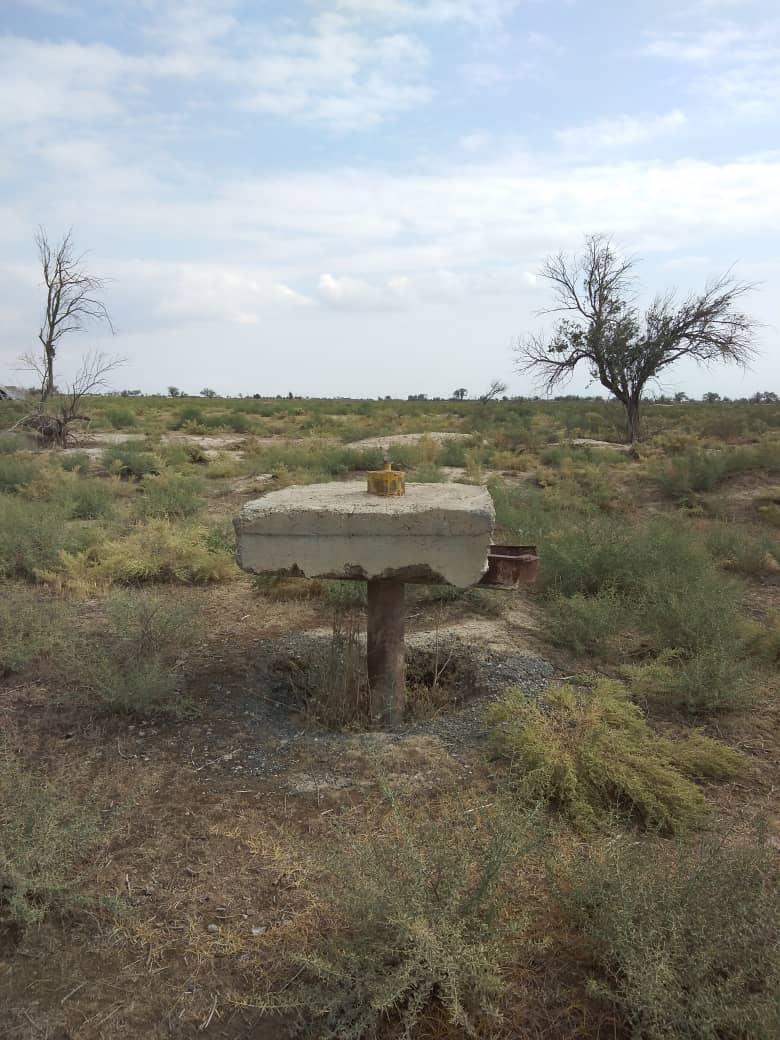


**Supplementary Fig.3**. An example of extensive land subsidence in the LUB, observed in a field trip in July 2020.


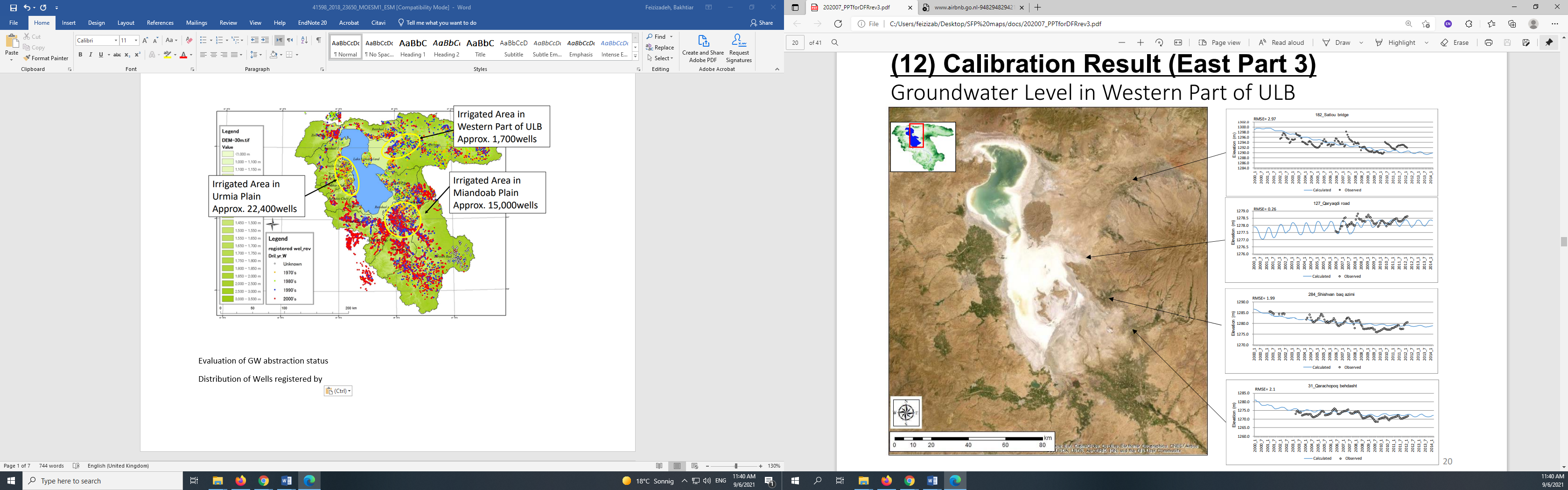
**Supplementary Fig.4.** Spatial distribution of the wells around the lake (source: water resources organizations in the study area and Urmia lake restoration program, <https://www.ulrp.ir/en/>)


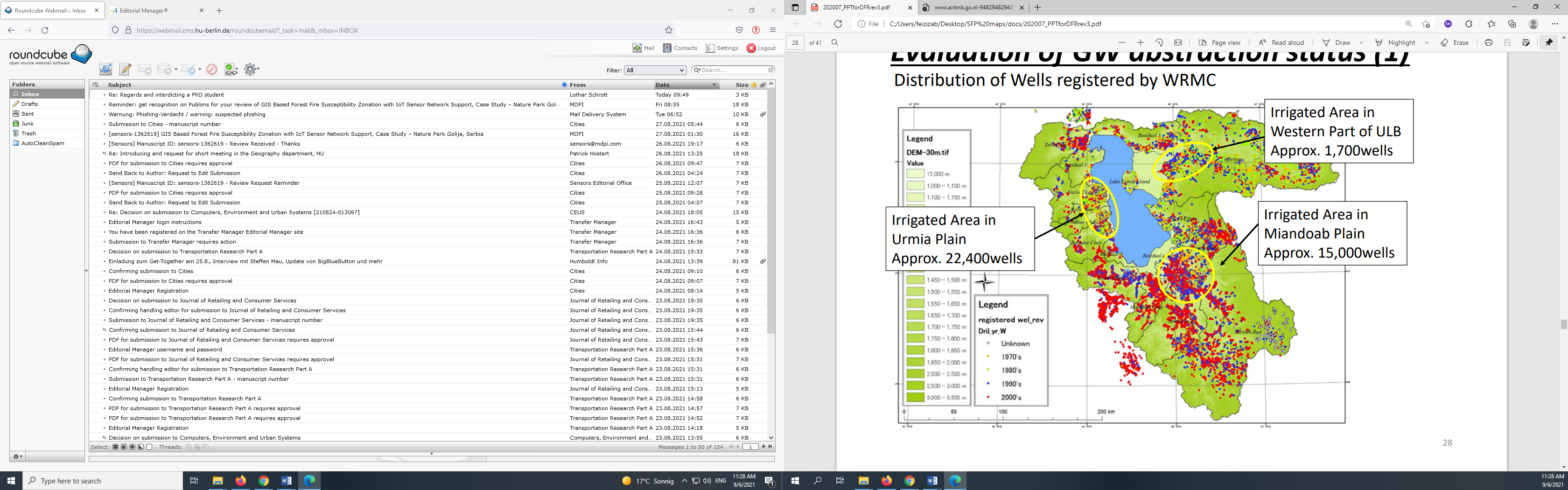


**Supplementary Fig.5.** Time series and trend analysis of extensive groundwater discharge in the nearby aquifers (Source: Urmia lake restoration program, https://www.ulrp.ir/en/)


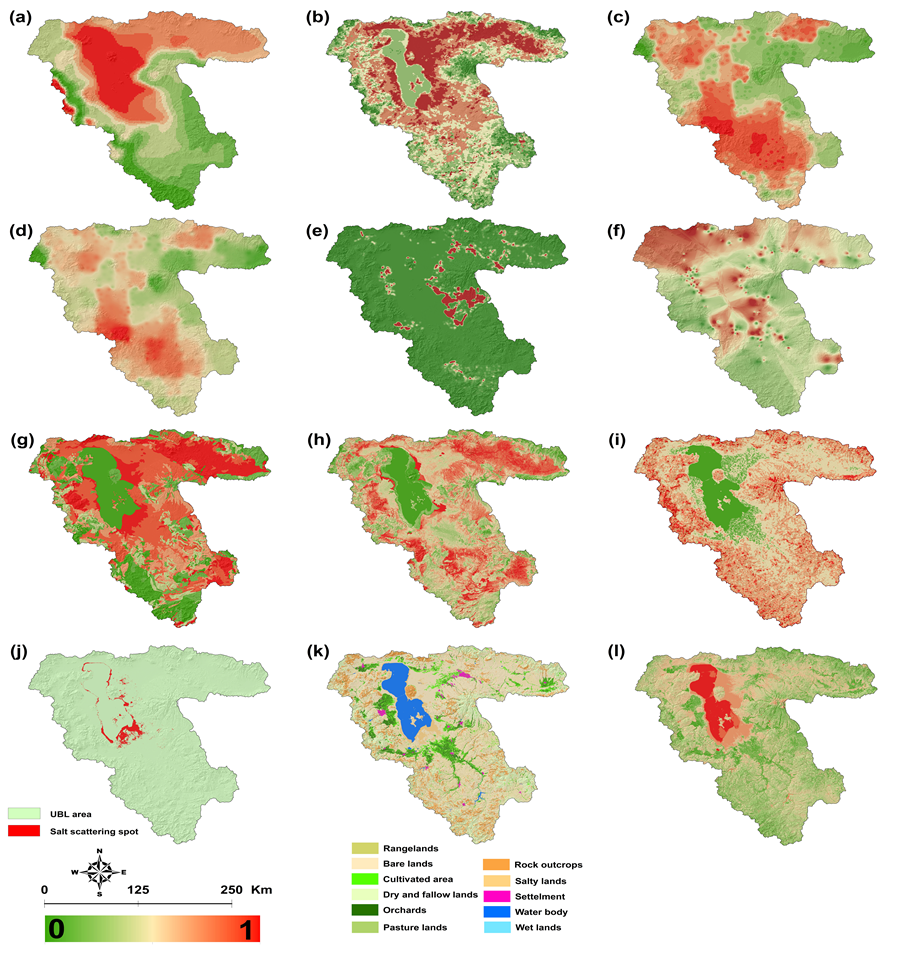


**Supplementary Fig.6.** spatial distribution of the selected indicators for food production mapping including: a) precipitation, b) temperature, c) humidity, d) sunshine hours, e) groundwater depth, f) water quality, g) soil degradation, h) soil fertility, i) soil texture, j) salt scattering spots, k) land use/cover, and I) soil depth (source: developed by the authors based on data obtained from Spatial Data Infrastructure (SDI) project of the study area )

| **Supplementary Table 1**. Annual average data for major crops produced in the Urmia lake basin and their contribution to food system of the country based on the annual report of the Ministry of Agriculture Jihad (https://maj.ir/) | | | |
| --- | --- | --- | --- |
|  | Crop | Tons | Contribution for the country % for |
| Crops products | Wheat | 270,120 | 7.8 |
|  | Oat | 253,217 | 5.2 |
|  | Lentils | 24,458 | 4.9 |
|  | Potato | 378,924 | 6.3 |
|  | Tomato | 42,235 | 13.4 |
|  | Pea | 47,580 | 6.8 |
|  | Onion | 124,500 | 8.7 |
|  | Bean | 52,060 | 2.4 |
|  | Alfa | 780,500 | 11.3 |
|  | Sugar beet | 455,000 | 13.8 |
| Horticultural products | Apricot | 11,250 | 7.8 |
|  | Appel | 995,000 | 21.4 |
|  | Grape | 485,200 | 11.1 |
|  | Peach & nectarines | 654,200 | 6.2 |
|  | Walnut | 34,525 | 8.7 |
|  | Cherries | 15,401 | 3.2 |
|  | Almond | 27,235 | 7.5 |
| livestock products | Red meat | 71,550 | 11.2 |
|  | White meat | 98,520 | 4.8 |
|  | Egg | 96,245 | 11.4 |
|  | Milk | 758,950 | 10.8 |
|  | Honey | 25,500 | 16.9 |
|  | Fish | 7,852 | 2.3 |
|  | Sum | 5,710,022 |  |

| **Supplementary Table 2**: *Difference* function and its respective default values for each category. The categories and respective values are part of the proposed method (for further details please Feizizadeh 2018 ^35^) | | | | |
| --- | --- | --- | --- | --- |
| Accuracy | Level of Confidence | Class map | | ICR % |
|  |  | Demand class | Alternative class |  |
| Confidence in classification | Very High Confidence in Classification (VHCC) | * |  | ≥ 90 |
|  | High Confidence in Classification (HCC) | * |  | ≥ 85 |
|  | Acceptable Confidence in Classification (ACC) |  | * | ≥ 80 |
|  | Reduced Confidence in Classification (RCC) | * |  | 80 ≤ |
|  | Very Reduced Confidence in Classification (VRCC) |  | * | 50 |
| Magnitude errors | Acceptable Error (AE) |  |  | 50 ≤ |
|  | High Error (HE) |  |  | ≥ 85 |
|  | Very High Error (VHE) |  |  | ≥ 90 |

| **Supplementary Table 3**. Results of the accuracy assessment and interpretation of confidence ratings (ICR) values for the integrated fuzzy object-based image analysis and deep learning method, which are applied for land use/cover change mapping (part 1). | | | | | | | | | | | | | | | | | | | | | | | | | | | | |
| --- | --- | --- | --- | --- | --- | --- | --- | --- | --- | --- | --- | --- | --- | --- | --- | --- | --- | --- | --- | --- | --- | --- | --- | --- | --- | --- | --- | --- |
| ICRs | Bare lands | | | | | | | Cultivated area | | | | | | | Dry and fallow | | | | | | | Orchards | | | | | | |
| Year | 1990 | 1995 | 2000 | 2005 | 2010 | 2015 | 2020 | 1990 | 1995 | 2000 | 2005 | 2010 | 2015 | 2020 | 1990 | 1995 | 2000 | 2005 | 2010 | 2015 | 2020 | 1990 | 1995 | 2000 | 2005 | 2010 | 2015 | 2020 |
| VHCC | 0.91 | 0.89 | 0.91 | 0.96 | 0.90 | 0.91 | 0.92 | 0.81 | 0.94 | 0.87 | 0.95 | 0.91 | 0.88 | 0.90 | 0.92 | 0.94 | 0.93 | 0.89 | 0.90 | 0.89 | 0.91 | 0.93 | 0.96 | 0.91 | 0.94 | 0.93 | 0.95 | 0.93 |
| HCC | 0.23 | 0.34 | 0.36 | 0.22 | 0.13 | 0.14 | 0.12 | 0.14 | 0.25 | 0.23 | 0.15 | 0.23 | 0.25 | 0.14 | 0.17 | 0.14 | 0.12 | 0.15 | 0.14 | 0.12 | 0.15 | 0.16 | 0.14 | 0.16 | 0.14 | 0.13 | 0.15 | 0.16 |
| ACC | 0.09 | 0.11 | 0.08 | 0.92 | 0.10 | 0.11 | 0.07 | 0.13 | 0.11 | 0.09 | 0.05 | 0.06 | 0.16 | 0.12 | 0.13 | 0.07 | 0.03 | 0.92 | 0.19 | 0.12 | 0.06 | 0.03 | 0.14 | 0.06 | 0.17 | 013 | 0.07 | 0.06 |
| RCC | 0.09 | 0.15 | 0.17 | 0.18 | 0.10 | 0.09 | 0.08 | 0.19 | 0.16 | 0.13 | 0.05 | 0.09 | 0.18 | 0.14 | 0.12 | 0.06 | 0.07 | 0.11 | 0.10 | 0.13 | 0.09 | 0.07 | 0.14 | 0.11 | 0.13 | 0.17 | 0.15 | 0.17 |
| VRCC | 0.02 | 0.03 | 0.06 | 0.07 | 0.03 | 0.08 | 0.02 | 0.09 | 0.08 | 0.06 | 0.05 | 0.04 | 0.06 | 0.05 | 0.07 | 0.04 | 0.03 | 0.02 | 0.04 | 0.09 | 0.02 | 0.03 | 0.08 | 0.06 | 0.06 | 0.05 | 0.05 | 0.03 |
| AE | 0.03 | 0.02 | 0.01 | 0.02 | 0.03 | 0.01 | 00.2 | 0.03 | 0.01 | 0.02 | 0.03 | 0.02 | 0.02 | 0.02 | 0.03 | 0.02 | 0.01 | 0.02 | 0.03 | 0.02 | 0.01 | 0.01 | 0.02 | 0.03 | 0.01 | 0.02 | 0.02 | 0.01 |
| HE | 0 | 0 | 0 | 0 | 0 | 0 | 0 | 0 | 0 | 0 | 0 | 0 | 0 | 0 | 0 | 0 | 0 | 0 | 0 | 0 | 0 | 0 | 0 | 0 | 0 | 0 | 0 | 0 |
| CHE | 0 | 0 | 0 | 0 | 0 | 0 | 0 | 0 | 0 | 0 | 0 | 0 | 0 | 0 | 0 | 0 | 0 | 0 | 0 | 0 | 0 | 0 | 0 | 0 | 0 | 0 | 0 | 0 |
| FSE % | 91.2 | 90.1 | 91.4 | 90.8 | 91.6 | 93.1 | 92.0 | 94.4 | 92.1 | 94.1 | 93.1 | 93.2 | 94.1 | 95.6 | 94.1 | 93.3 | 92.8 | 91.9 | 93.2 | 91.6 | 95.2 | 93.9 | 94.3 | 94.2 | 92.1 | 94.5 | 95.4 | 94.9 |

| **Supplementary Table 3**. Results of the accuracy assessment and interpretation of confidence ratings (ICR) values for the integrated fuzzy object-based image analysis and deep learning method, which are applied for land use/cover change mapping (part 2). | | | | | | | | | | | | | | | | | | | | | | | | | | | | |
| --- | --- | --- | --- | --- | --- | --- | --- | --- | --- | --- | --- | --- | --- | --- | --- | --- | --- | --- | --- | --- | --- | --- | --- | --- | --- | --- | --- | --- |
| ICRs | Pasture lands | | | | | | | Rangelands | | | | | | | Rock output | | | | | | | Salty lands | | | | | | |
| Year | 1990 | 1995 | 2000 | 2005 | 2010 | 2015 | 2020 | 1990 | 1995 | 2000 | 2005 | 2010 | 2015 | 2020 | 1990 | 1995 | 2000 | 2005 | 2010 | 2015 | 2020 | 1990 | 1995 | 2000 | 2005 | 2010 | 2015 | 2020 |
| VHCC | 0.95 | 0.92 | 0.93 | 0.92 | 0.91 | 0.93 | 0.92 | 0.86 | 0.93 | 0.89 | 0.91 | 0.89 | 0.93 | 0.91 | 0.94 | 0.92 | 0.95 | 0.94 | 0.92 | 0.89 | 0.92 | 0.91 | 0.95 | 0.94 | 0.93 | 0.94 | 0.95 | 0.97 |
| HCC | 0.02 | 0.21 | 0.09 | 0.13 | 0.13 | 0.12 | 0.12 | 0.14 | 0.25 | 0.23 | 0.15 | 0.23 | 0.25 | 0.14 | 0.17 | 0.14 | 0.12 | 0.15 | 0.14 | 0.12 | 0.15 | 0.16 | 0.14 | 0.16 | 0.14 | 0.13 | 0.15 | 0.16 |
| ACC | 0.01 | 0.14 | 0.12 | 0.92 | 0.10 | 0.10 | 0.07 | 0.13 | 0.11 | 0.09 | 0.05 | 0.06 | 0.12 | 0.11 | 0.13 | 0.07 | 0.03 | 0.92 | 0.19 | 0.12 | 0.06 | 0.03 | 0.14 | 0.06 | 0.17 | 013 | 0.07 | 0.06 |
| RCC | 0.05 | 0.14 | 0.11 | 0.18 | 0.10 | 0.09 | 0.04 | 0.14 | 0.16 | 0.12 | 0.05 | 0.09 | 0.12 | 0.14 | 0.12 | 0.06 | 0.06 | 0.11 | 0.10 | 0.13 | 0.09 | 0.07 | 0.14 | 0.11 | 0.13 | 0.17 | 0.15 | 0.12 |
| VRCC | 0.01 | 0.02 | 0.02 | 0.07 | 0.03 | 0.08 | 0.02 | 0.09 | 0.08 | 0.06 | 0.05 | 0.04 | 0.06 | 0.05 | 0.02 | 0.04 | 0.03 | 0.02 | 0.04 | 0.09 | 0.02 | 0.03 | 0.08 | 0.06 | 0.06 | 0.05 | 0.05 | 0.03 |
| AE | 0.02 | 0.0 | 0.03 | 0.02 | 0.03 | 0.01 | 00.2 | 0.03 | 0.01 | 0.02 | 0.02 | 0.01 | 0.03 | 0.02 | 0.01 | 0.02 | 0.01 | 0.02 | 0.03 | 0.02 | 0.01 | 0.01 | 0.02 | 0.03 | 0.01 | 0.02 | 0.02 | 0.01 |
| HE | 0 | 0 | 0 | 0 | 0 | 0 | 0 | 0 | 0 | 0 | 0 | 0 | 0 | 0 | 0 | 0 | 0 | 0 | 0 | 0 | 0 | 0 | 0 | 0 | 0 | 0 | 0 | 0 |
| CHE | 0 | 0 | 0 | 0 | 0 | 0 | 0 | 0 | 0 | 0 | 0 | 0 | 0 | 0 | 0 | 0 | 0 | 0 | 0 | 0 | 0 | 0 | 0 | 0 | 0 | 0 | 0 | 0 |
| FSE % | 94.1 | 91.3 | 92.3 | 90.8 | 92.7 | 92.1 | 92.0 | 90.9 | 93.1 | 90.1 | 92.9 | 91.7 | 94.5 | 95.6 | 94.1 | 93.3 | 91.6 | 91.9 | 93.2 | 90.6 | 93.2 | 94.2 | 95.3 | 94.2 | 92.1 | 94.5 | 92.3 | 94.9 |

| **Supplementary Table 3**. Results of the accuracy assessment and interpretation of confidence ratings (ICR) values for the integrated fuzzy object-based image analysis and deep learning method, which are applied for land use/cover change mapping (part 3). | | | | | | | | | | | | | | | | | | | | | |
| --- | --- | --- | --- | --- | --- | --- | --- | --- | --- | --- | --- | --- | --- | --- | --- | --- | --- | --- | --- | --- | --- |
| ICRs | Settlement | | | | | | | Water body | | | | | | | Wet Lands | | | | | | |
| Year | 1990 | 1995 | 2000 | 2005 | 2010 | 2015 | 2020 | 1990 | 1995 | 2000 | 2005 | 2010 | 2015 | 2020 | 1990 | 1995 | 2000 | 2005 | 2010 | 2015 | 2020 |
| VHCC | 0.94 | 0.92 | 0.93 | 0.95 | 0.92 | 0.93 | 0.94 | 0.92 | 0.94 | 0.95 | 0.95 | 0.95 | 0.97 | 0.96 | 0.95 | 0.94 | 0.93 | 0.89 | 0.94 | 0.96 | 0.96 |
| HCC | 0.13 | 0.21 | 0.12 | 0.05 | 0.13 | 0.16 | 0.10 | 0.11 | 0.13 | 0.02 | 0.15 | 0.13 | 0.02 | 0.11 | 0.12 | 0.15 | 0.11 | 0.10 | 0.13 | 0.11 | 0.13 |
| ACC | 0.04 | 0.09 | 0.04 | 0.92 | 0.12 | 0.08 | 0.04 | 0.02 | 0.10 | 0.11 | 0.05 | 0.06 | 0.16 | 0.12 | 0.13 | 0.07 | 0.03 | 0.92 | 0.19 | 0.12 | 0.06 |
| RCC | 0.08 | 0.12 | 0.11 | 0.12 | 0.10 | 0.09 | 0.02 | 0.11 | 0.12 | 0.11 | 0.02 | 0.08 | 0.18 | 0.14 | 0.12 | 0.06 | 0.06 | 0.11 | 0.10 | 0.09 | 0.07 |
| VRCC | 0.03 | 0.10 | 0.02 | 0.05 | 0.03 | 0.08 | 0.02 | 0.09 | 0.08 | 0.06 | 0.05 | 0.04 | 0.06 | 0.05 | 0.07 | 0.04 | 0.03 | 0.02 | 0.04 | 0.09 | 0.02 |
| AE | 0.01 | 0.02 | 0.03 | 0.02 | 0.03 | 0.01 | 00.2 | 0.03 | 0.01 | 0.02 | 0.03 | 0.02 | 0.02 | 0.02 | 0.03 | 0.02 | 0.01 | 0.02 | 0.03 | 0.02 | 0.01 |
| HE | 0 | 0 | 0 | 0 | 0 | 0 | 0 | 0 | 0 | 0 | 0 | 0 | 0 | 0 | 0 | 0 | 0 | 0 | 0 | 0 | 0 |
| CHE | 0 | 0 | 0 | 0 | 0 | 0 | 0 | 0 | 0 | 0 | 0 | 0 | 0 | 0 | 0 | 0 | 0 | 0 | 0 | 0 | 0 |
| FSE % | 92.2 | 90.1 | 91.4 | 94.6 | 92.1 | 91.4 | 93.6 | 92.4 | 93.1 | 95.1 | 96.2 | 94.2 | 96.1 | 95.6 | 94.1 | 93.3 | 92.8 | 91.9 | 93.2 | 91.6 | 95.2 |

| **Supplementary Table 4.** Compression matrix of the fuzzy analytical network for criteria weighting | | | | | | | | | | | | | |
| --- | --- | --- | --- | --- | --- | --- | --- | --- | --- | --- | --- | --- | --- |
| Criteria | | Precipitation | Temperature | Humidity | Sunshine hours | Groundwater depth | Water quality | Soil degradation | Soil fertility | Soil texture | Salt scattering spots | Land use/cover | Soil depth |
| Precipitation | | 0.07731 | 0.07731 | 0.07731 | 0.07731 | 0.07731 | 0.07731 | 0.07731 | 0.07731 | 0.07731 | 0.07731 | 0.07731 | 0.07731 |
| Temperature | | 0.09024 | 0.09024 | 0.09024 | 0.09024 | 0.09024 | 0.09024 | 0.09024 | 0.09024 | 0.09024 | 0.09024 | 0.09024 | 0.09024 |
| Humidity | | 0.11431 | 0.11431 | 0.11431 | 0.11431 | 0.11431 | 0.11431 | 0.11431 | 0.11431 | 0.11431 | 0.11431 | 0.11431 | 0.11431 |
| Sunshine hours | | 0.11095 | 0.11095 | 0.11095 | 0.11095 | 0.11095 | 0.11095 | 0.11095 | 0.11095 | 0.11095 | 0.11095 | 0.11095 | 0.11095 |
| Groundwater depth | | 0.08678 | 0.08678 | 0.08678 | 0.08678 | 0.08678 | 0.08678 | 0.08678 | 0.08678 | 0.08678 | 0.08678 | 0.08678 | 0.08678 |
| Water quality | | 0.07287 | 0.07287 | 0.07287 | 0.07287 | 0.07287 | 0.07287 | 0.07287 | 0.07287 | 0.07287 | 0.07287 | 0.07287 | 0.07287 |
| Soil degradation | | 0.01475 | 0.01475 | 0.01475 | 0.01475 | 0.01475 | 0.01475 | 0.01475 | 0.01475 | 0.01475 | 0.01475 | 0.01475 | 0.01475 |
| Soil fertility | | 0.08685 | 0.08685 | 0.08685 | 0.08685 | 0.08685 | 0.08685 | 0.08685 | 0.08685 | 0.08685 | 0.08685 | 0.08685 | 0.08685 |
| Soil texture | | 0.08579 | 0.08579 | 0.08579 | 0.08579 | 0.08579 | 0.08579 | 0.08579 | 0.08579 | 0.08579 | 0.08579 | 0.08579 | 0.08579 |
| Salt scattering spots | | 0.08675 | 0.08675 | 0.08675 | 0.08675 | 0.08675 | 0.08675 | 0.08675 | 0.08675 | 0.08675 | 0.08675 | 0.08675 | 0.08675 |
| Land use/cover | | 0.08521 | 0.08521 | 0.08521 | 0.08521 | 0.08521 | 0.08521 | 0.08521 | 0.08521 | 0.08521 | 0.08521 | 0.08521 | 0.08521 |
| Soil depth | | 0.08818 | 0.08818 | 0.08818 | 0.08818 | 0.08818 | 0.08818 | 0.08818 | 0.08818 | 0.08818 | 0.08818 | 0.08818 | 0.08818 |
|  |  |  |  |  |  |  |  |  |  |  |  |  |  |

1. Corresponding author: [Feizizab@hu-berlin.de](mailto:Feizizab@hu-berlin.de) [↑](#footnote-ref-1)
